# Novel Meiotic miRNAs and Indications for a Role of PhasiRNAs in Meiosis

**DOI:** 10.3389/fpls.2016.00762

**Published:** 2016-06-02

**Authors:** Stefanie Dukowic-Schulze, Anitha Sundararajan, Thiruvarangan Ramaraj, Shahryar Kianian, Wojciech P. Pawlowski, Joann Mudge, Changbin Chen

**Affiliations:** ^1^Department of Horticultural Science, University of Minnesota, St. Paul MN, USA; ^2^National Center for Genome Resources, Santa Fe NM, USA; ^3^Cereal Disease Laboratory, United States Department of Agriculture – Agricultural Research Service, St. Paul MN, USA; ^4^Section of Plant Biology, School of Integrative Plant Science, Cornell University, Ithaca NY, USA

**Keywords:** meiosis, meiocytes, small RNA, phasiRNA, DNA methylation, maize, sRNA-seq, bisulfite sequencing

## Abstract

Small RNAs (sRNA) add additional layers to the regulation of gene expression, with siRNAs directing gene silencing at the DNA level by RdDM (RNA-directed DNA methylation), and micro RNAs (miRNAs) directing post-transcriptional regulation of specific target genes, mostly by mRNA cleavage. We used manually isolated male meiocytes from maize (*Zea mays*) to investigate sRNA and DNA methylation landscapes during zygotene, an early stage of meiosis during which steps of meiotic recombination and synapsis of paired homologous chromosomes take place. We discovered two novel miRNAs from meiocytes, zma-MIR11969 and zma-MIR11970, and identified putative target genes. Furthermore, we detected abundant phasiRNAs of 21 and 24 nt length. PhasiRNAs are phased small RNAs which occur in 21 or 24 nt intervals, at a few hundred loci, specifically in male reproductive tissues in grasses. So far, the function of phasiRNAs remained elusive. Data from isolated meiocytes now revealed elevated DNA methylation at phasiRNA loci, especially in the CHH context, suggesting a role for phasiRNAs in *cis* DNA methylation. In addition, we consider a role of these phasiRNAs in chromatin remodeling/dynamics during meiosis. However, this is not well supported yet and will need more additional data. Here, we only lay out the idea due to other relevant literature and our additional observation of a peculiar GC content pattern at phasiRNA loci. Chromatin remodeling is also indicated by the discovery that histone genes were enriched for sRNA of 22 nt length. Taken together, we gained clues that lead us to hypothesize sRNA-driven DNA methylation and possibly chromatin remodeling during male meiosis in the monocot maize which is in line with and extends previous knowledge.

## Introduction

Examining gene expression provides important information to understand how processes in distinct cell types and stages during development are orchestrated. Gene expression is a multi-layer process, in which the DNA sequence of a gene provides the blueprint for the synthesis of a functional gene product that can be a protein, a structural RNA, or another non-coding RNA. Current sequencing technologies enable large-scale analysis of RNA quantification, but mRNA levels are not sufficient to completely reflect final levels of gene products. In the case of protein products, global analysis of protein content is of course preferable and can substantially deviate from mere mRNA levels (Smits et al., 2014), but large-scale proteomics still lag behind transcriptomics. Also, not all gene products are proteins, and gaining more information on other aspects, and on transcriptional and post-transcriptional regulation, can partly be achieved by examining non-coding RNAs, including small RNAs.

Small non-coding RNAs in plants belong to the main categories of either micro RNA (miRNA) or small interfering RNA (siRNA), while animals have an additional, reproduction-specific category, PIWI-interacting RNA (piRNA; reviewed in: Chen, 2009; Arikit et al., 2013). Generally, miRNAs have important roles in down-regulating gene expression especially in plant development (Jones-Rhoades and Bartel, 2004; Borges et al., 2011; reviewed in: Mallory and Vaucheret, 2006), while siRNAs are primarily targeting and silencing exogenous sequences like transposable elements (TEs), transgenes and viruses (reviewed in: Baulcombe, 2004). Transacting siRNA (tasiRNA) and phased siRNA (phasiRNA) are plant-specific siRNA subcategories which are both characterized by being phased, i.e., occurring in intervals of 21 or 24 nucleotides. The tasiRNA loci are few in number (four TAS families in *Arabidopsis*; Chen et al., 2007) but have identified important target genes including Auxin Responsive Factors (ARFs, involved in growth and development), pentatricopeptide repeat proteins (PPRs, involved in RNA processing) and nucleotide-binding, leucine-rich repeat proteins (NB-LRRs, involved in disease defense; Allen et al., 2005; Adenot et al., 2006; Fahlgren et al., 2006; Howell et al., 2007; Zhai et al., 2011; Xia et al., 2013). PhasiRNAs are also phased but have no identified target genes and occur in far higher numbers, specifically in the reproductive tissue in early anther development in grasses (Johnson et al., 2009; Song et al., 2012; Zhai et al., 2015). Thus, the existence and abundance of phasiRNAs during male gametogenesis in plants points to a unique function for these sRNA species, but the function remained elusive so far.

The three common effects of small non-coding RNAs are cleavage (slicing) of target mRNA, inhibition of mRNA translation, and chromatin modification (reviewed in: Bonnet et al., 2006; Chen, 2009; Borges and Martienssen, 2015). In plants, most sRNA are siRNA that stem from repetitive-rich regions and regulate silencing of chromatin – a notable exception to this is sRNA from the moss *Physcomitrella patens* which is most abundant in miRNAs (Coruh et al., 2015). Plant miRNAs appear to differ from their animal counterparts in the extent of complementarity to their target genes: While animal miRNAs usually have short seed regions including nucleotides 2–7 (Zhou et al., 2009; Fabian and Sonenberg, 2012; reviewed in: Lewis et al., 2003; Bartel, 2009), plant miRNAs frequently have near-perfect complementarity with their targets, thereby having fewer targets (reviewed in Voinnet, 2009). One hypothesis proposed that the degree of miRNA complementarity to their targets determines the mode of action – mRNA cleavage in the case of high complementarity, and translational repression in the case of multiple mismatches (Hutvágner and Zamore, 2002). The high complementarity in plants results thus in mostly slicing (Mallory et al., 2004; reviewed in: Jones-Rhoades et al., 2006). However, translational inhibition has since been described as more widespread (Brodersen et al., 2008), and it was suggested that the criterion for identifying plant miRNA targets should be extended to mismatched targets (Dugas and Bartel, 2008).

MiRNAs frequently target transcription factors for stage transitions in plant development, and defects in miRNA expression or sequence alteration can lead to visible developmental defects and phenotypic changes (Jones-Rhoades and Bartel, 2004; Gou et al., 2011; reviewed in: Mallory and Vaucheret, 2006). MiR156 and MiR159 for example target SPL and MYB transcription factors, and their overexpression can cause late flowering and male sterility (Achard et al., 2004; Millar and Gubler, 2005; Schwab et al., 2005). Other genes involved in reproductive development that are regulated by miRNAs are ARF6 and ARF8 (regulating anther and ovule development, including anther dehiscence, targeted by miR167), homeotic class C genes (defining flower whorl architecture, targeted by miR169), and the *APETALA2* homeotic gene and *TOE1* (involved in flower whorl architecture, spikelet determination, and flowering time, targeted by miR172; Aukerman and Sakai, 2003; Wu et al., 2006; Cartolano et al., 2007). Thus, miRNAs are an important component of regulating gene expression during reproductive development. All these miRNAs are present in both dicot plants and grasses, and their initial identification and characterization in mostly dicot plants have been followed by studies in grasses, including maize, rice, barley, and oats (reviewed in: Luo et al., 2013).

Other types of sRNA also play crucial roles in plant reproductive development. An intriguing phenomenon in mature pollen was discovered with single cell type techniques in *Arabidopsis*: In pollen, transposons are activated in the vegetative nucleus, causing TE-derived 21 nt siRNAs which, in turn, accumulate in the generative nucleus where they regulate DNA methylation at TE loci for repression (Slotkin et al., 2009; reviewed in: Borges et al., 2011). A similar reactivation and subsequent chromatin silencing of TEs occurs in ovules, in companion cells and megaspore mother cells, respectively (Olmedo-Monfil et al., 2010). Small RNA data from isolated cells that are in early stages of meiosis has not been reported yet and might shed some light on the function of phasiRNAs, and on the involvement of sRNA in the regulation of unique processes during pairing and synapsis of homologous chromosomes and meiotic recombination. We address these question here by using isolated populations of maize (*Zea mays*) male meiocytes during zygotene for sRNA sequencing and bisulfite-conversion sequencing.

## Materials and Methods

### Sample Preparation and Sequencing

In brief, maize (*Zea mays*) plants of the inbred lines B73 and CML228 were grown in the greenhouse, and reproductive samples collected at the time of zygotene during male meiosis. Meiocytes were isolated via a microcapillary collection method (Chen and Retzel, 2013; Dukowic-Schulze et al., 2014b), and meiocytes and corresponding anthers from at least five plants were pooled per sample. Three 2-week-old seedlings were pooled and used as vegetative control sample. Our previous RNA-seq study (Dukowic-Schulze et al., 2014a) was based on two replicates, whereas we conducted our current sRNA study with one sample each of CML228 and B73 to gain clues on similarity and differences between inbred lines without overstraining the requirement for isolating meiocytes, which is a tedious and technically demanding procedure. Noteworthy is that we used the extracted total RNA from the CML228 sample for both small RNA and mRNA library preparation in parallel, by separating them on a gel. Kits used were (i) RNAqueous Micro Kit from Ambion for extraction of total RNA from small sample amounts, (ii) Qubit RNA BR Assay Kit from Invitrogen for measuring RNA, (iii) TruSeq or TruSeq Small RNA protocol from Illumina Technologies for RNA library preparation including a polyA selection step in the former. An Illumina HiSeq2000 machine was used to generate single-end 1x50 bp reads for mRNA-seq, and single-end 1x36 bp reads for sRNA-seq.

Accordingly, samples of B73 were also processed for bisulfite sequencing, starting from extracted chromatin via bisulfite conversion with the EZ DNA Methylation-Gold Kit from the Zymo Research Corporation, and ending in 2x100 bp paired-end reads from an Illumina HiSeq2000 machine. All steps of sample preparation, sequencing, library preparation are described in detail in Dukowic-Schulze et al. (2014b).

### Read Alignment

Detailed workflow and post-processing of the raw reads is described in Dukowic-Schulze et al. (2014b). For small RNA reads, GSNAP (**G**enomic **S**hort-read **N**ucleotide **A**lignment **P**rogram) was used for alignment (Wu and Nacu, 2010) to the reference genome, allowing no more than five matching loci with the same score for each read in the genome. Later, we allowed up to 85 matching loci, the highest value possible for successfully computing all samples. In order to perform sRNA analysis with ShortStack (Axtell, 2013), the small RNA-seq data was aligned with its accompanying alignment tool “butter” (**B**owtie **ut**ilizing i**t**erative plac**e**ment of **r**epetitive small RNAs; Johnson et al., 2016). Consistent with our RNA-seq data, we used RefGen_v2 of the B73 maize reference genome for all alignments. In the case of bisulfite-converted DNA from B73 samples, methylated cytosine and their contexts (CG, CHG, CHH) were extracted with Bismark Bisulfite Mapper (Krueger and Andrews, 2011), as described in Dukowic-Schulze et al. (2014b). For that, paired-end FASTQ reads were used as input with quality encoding set to PHRED 33 and BOWTIE2 was the choice of aligner. For alignments, the maximum number of mismatches permitted was set to 1 bp, seed length was set to 28 bp and minimum and maximum insert size for valid paired-end alignments was set to 0 and 500 bp, respectively. SAMtools was used, and the alignment output was in SAM format. To make the data suitable for downstream analysis, BED (Browser Extensible Data) files of the methylation percentage per 100 bp tile were generated for each sample and context by using a database and tailored database queries.

### sRNA Analysis

SAMTools (Li et al., 2009) was used via the Unix command line to extract data from BAM alignment files for the production of Excel graphs for size distribution, read mapping, and genomic feature overlap. Aligned reads were visualized with IGV (Integrative Genomics Viewer, Broad Institute; Robinson et al., 2011), with improved calculation and displaying facilitated by created TDF files. Exaggerated background read reduction for diverse downstream analyses, including phasiRNA loci determination, was achieved by removing reads from any loci with less than two RPM (reads per million). Coverage plots and correlation heat map were computed using BEDTOOLS (Quinlan, 2014) and graphed using the R Statistical Program. Importantly, instances with no methylation information need to be ignored, and not treated as 0%. Rows with “.” were thus removed by “grep” after “bedtools map”, before “bedtools groupby”. Coverage plots are used to average e.g., DNA methylation percentage or the proportion of a feature presence over multiple loci. For example, if 100 loci of interest are analyzed for their overlap with annotated genes, and 80 of them do overlap, the coverage plot *y*-value at the start or mid of the loci is 80%; however, since not all loci or hit genes have the same length, the percentage decreases when proceeding on the *y*-axis. For effects of sRNAs in trans, differential expression of miRNAs was tackled by generating read counts for miRBase (Griffiths-Jones, 2006) entries for maize with our initial GSNAP alignment, and also by running ShortStack analysis with a flagfile which included known miRNA gene loci. BLASTN (task blastn-short) algorithmfromthe NCBI BLAST+ suite (Camacho et al., 2009) was run via Unix Command Line to check whole sRNA cluster regions annotated by ShortStack as miRNA against miRNAs listed in the miRBase database. The resulting short miRNA sequences were checked directly online against miRBase with SSEARCH parameters. Target gene prediction for putative miRNAs was performed with psRNATarget (Dai and Zhao, 2011). For the effect of sRNAs in *cis*, all genes overlapping sRNA clusters identified by ShortStack were analyzed for overlaps between samples via BioVenn (Hulsen et al., 2008) and Venny (Oliveros, 2007), and subjected to GO (Gene Ontology) annotation via AgriGO (Du et al., 2010). Examination of differentially expressed sRNA loci was done using ShortStack in count mode (Axtell, 2013), and the Bioconductor DEseq package for R (Anders and Huber, 2010).

## Results

### Expression Profiles of sRNA Pathway Components and sRNAs in Meiocytes

**FIGURE 1:**
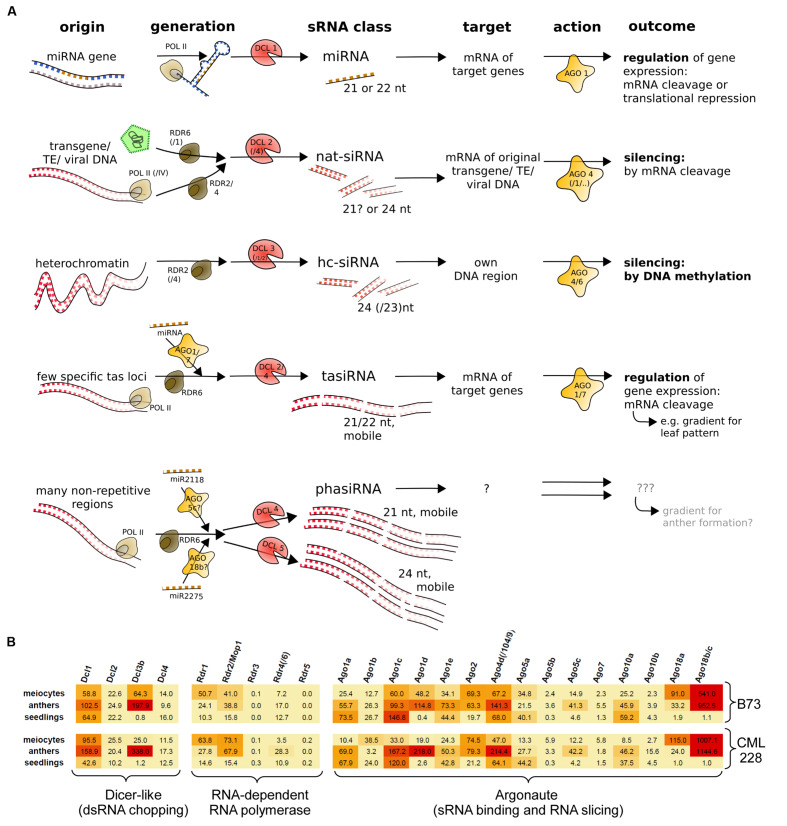
**sRNA pathway components.** **(A)** Simplified overview of typical sRNA pathways and components in plants. Shown are three primary sRNA types (miRNA, nat-siRNA, hc-siRNA) and two secondary sRNAs (tasiRNA and phasiRNA). **(B)** mRNA expression levels of maize sRNA pathway components, illustrated by color-coding (red = high level, orange = medium level, yellow = low expression).

Small RNAs differ in their biogenesis and function, and originally there was a clear distinction between miRNAs which act *in trans* and siRNAs (i.e., nat-siRNA and hc-siRNAs) which act *in cis* (**Figure 1A**). Plant-specific secondary sRNAs, i.e., tasiRNAs and reproductive phasiRNAs, added a new category – they have no discerned targets or targets *in trans* and are triggered by miRNAs (**Figure 1A**). We previously reported the characterization of mRNA-seq data obtained from isolated maize meiocytes via polyA selection and Illumina sequencing (Dukowic-Schulze et al., 2014a), and now have exploited this data further to examine the expression of genes in sRNA pathways. The maize inbred line B73 we study is widely used in genetic studies as well as for hybrid breeding, and an assembled reference genome is available. We extended our analysis by adding the transcriptome of CML228, a tropical maize inbred line. Here, we looked at the normalized expression of the putative maize genes for Dicer-like proteins, Argonaute proteins and RNA-dependent RNA polymerases as mainly identified by Qian et al. (2011). The two instances where isolated meiocytes showed the highest gene expression between the examined samples are *Rdr1* and *Ago18a* (**Figure 1B**). In far more cases, the highest expression is in whole anthers followed by meiocytes, e.g., for *Dcl3b, Rdr2/Mop1, Ago1d, Ago4d/Ago104/Ago9, Ago5c*, and especially *Ago18b/c* which is almost exclusively specific to the male reproductive organ, confirmed by our own data from the second inbred line, CML228 (**Figure 1B**), as well as previous reports for B73 easily visualized via the eFPBrowser^1^ (data from Sekhon et al., 2011; Downs et al., 2013).

**FIGURE 2:**
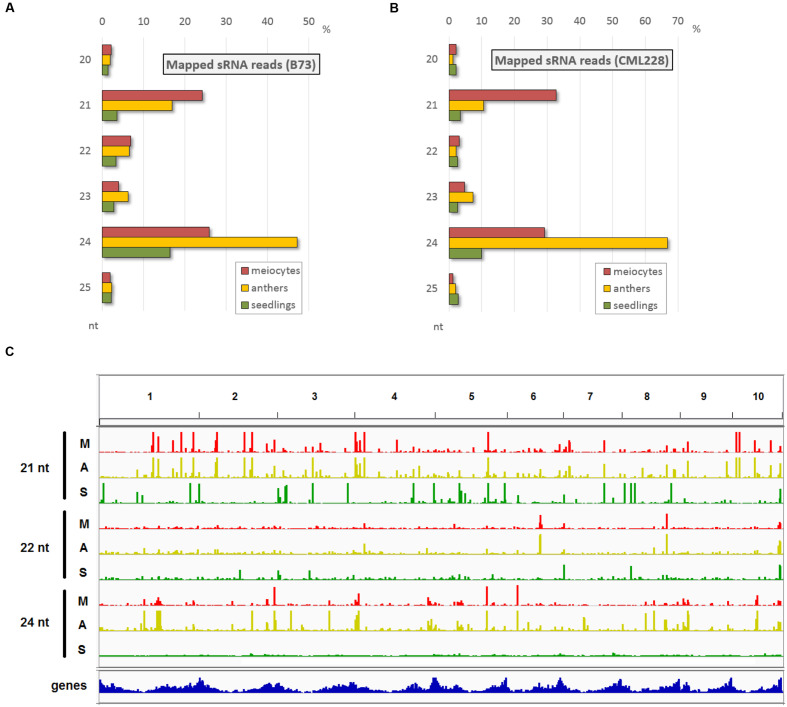
**Main sRNA populations during early maize meiosis (zygotene stage).** **(A,B)** sRNA size distribution in the most relevant range (20–25 nt) for B73 **(A)** and CML228 **(B)**. **(C)** B73 sRNA distribution across the 10 maize chromosomes, for 21, 22, 24 nt length per sample (M = meiocytes, in red; A = anthers, in yellow; S = seedlings, in green). Depicted via IGV.

Since, we had special RNA pathway components highly expressed in male reproductive tissue and we wanted to have a more comprehensive overview on the cellular events during early meiosis, we generated sRNA-seq data for the same samples as used before, i.e., isolated male maize meiocytes during zygotene (the prophase I sub-stage during which recombination events take place), corresponding whole anthers, and whole 2-week-old seedlings. sRNA reads in the range of 15–36 nt were aligned, and as expected, the majority are of 21, 22, and 24 nt lengths (**Figures 2A,B**) – the known functional sRNAs as summarized in **Figure 1A**. Interestingly, the proportion of these relevant reads in meiocyte and anther samples exceeded the ones in seedling samples by far in both inbred lines accordingly (**Figures 2A,B**). While the zygotene meiocyte samples have the most reads of 21 nt lengths, the zygotene anther samples have the highest proportion of 24 nt reads (**Figures 2A,B**). Read distribution per sample and nucleotide size across all ten chromosomes reveals a similar pattern between meiocyte and anther samples where clusters tend to be at the same positions, and illustrates the broader distribution in especially 24 nt reads in the case of the seedling sample (**Figure 2C**).

Small RNA reads were examined for overlap with annotated genomic features. Samples from both inbred lines show similar trends, and if considering all aligned reads, overlap with genes and TEs have similar proportions (**Supplementary Figure S1A**) while the major proportion of sRNA reads aligned to genomic regions with no annotations in the databases we used. However, when considering only sRNAs at positions with a more biologically relevant ≥2 RPM, the proportion of overlap with TEs is the highest, followed by unannotated regions, followed by genes (**Supplementary Figure S1B**). Detailing the TE overlap by distinguishing between TE superfamilies and sRNA nucleotide length, and restricting the data from total sRNA reads to sRNA loci with ≥2 RPM emphasizes the similarity between isolated meiocytes and whole anthers in contrast to seedlings (**Supplementary Figure S1C**). There are fewer loci ≥2 RPM in seedlings than for meiocytes and anthers (for 21 nt: 83/73/7, for 22 nt: 46/47/11, for 24 nt: 283/356/149 for meiocytes/anthers/seedlings, respectively), and especially the higher proportion of hAT superfamily overlap for 24 nt sRNAs in seedlings, while being absent in the 22 nt sRNA seedling data, is of note (**Supplementary Figure S1C**).

Taken together, single-cell type sequencing data of RNA transcripts and small non-coding RNAs during early meiosis highlight the difference to vegetative tissue, and reveal that meiocytes shape the sRNA profile of whole anthers (**Figure 2C**) whereas only few sRNA pathway components are up-regulated in isolated meiocytes vs. whole anthers (**Figure 1B**).

### sRNA Mapping

Although many guidelines and software tools exist for mapping and interpreting mRNA-seq data, sRNA-seq data analysis tools still lag behind. We thus used different approaches for the initial step of read alignment since this can impact the outcomes substantially. The conservative use of the GSNAP alignment algorithm (Wu and Nacu, 2010), allowing a read to map to a maximum of five different locations in the genome, resulted in 51.76 and 38.69% aligned reads for meiocytes and seedlings, respectively (**Table 1**). Adjusting the maximum ambiguity parameter to the highest setting possible on our hardware (up to 85 multiple locations) increased the alignment rate drastically (to 63.31 and 71.95%). Notable is the difference in the amount of increase in meiocytes vs. seedlings (**Table 1**; 11.55 percentage points vs. 33.26 percentage points; or when calculated in percent increase 22.31 vs. 85.96%). The high increase in the alignment rate in seedlings is due to more multi-mapping reads, presumably TEs.

**Table 1:** Rate of successfully aligned reads.

	Meiocytes (M)	Seedlings (S)	Ratio M/S
Alpheus pipeline (5max_amb)	18,997,952 (51.76%)	31,796,919 (38.69%)	1.34
Alpheus pipeline (85max_amb)	23,236,071 (63.31%)	59,125,132 (71.95%)	0.88
Butter aligner (ShortStack)	31,249,932 (85.14%)	67,420,822 (82.05%)	1.04
Total reads	36,703,499	82,175,280	


*Comparison of three different approaches for alignment of sRNA data to the B73 reference genome. Parameter adjustment 5max_amb and 85max_amb means only five allowed multiple mapping locations vs. 85.*

As an alternative to the aligner “GSNAP” we also used the aligner “butter” (**B**owtie **ut**ilizing i**t**erative plac**e**ment of **r**epetitive small RNAs; Johnson et al., 2016), which was developed specifically for sRNA alignment. This improved the alignment rate further to 85.14 and 82.05%, and resulted in a more balanced meiocytes-to-seedlings ratio (**Table 1**). The “butter” alignment algorithm allows up to 1000 ambiguous regions when read placement can be guided by density, up to three ambiguous regions if not (Johnson et al., 2016). Since we were most interested in sRNAs that align uniquely, we mainly used the data from the conservative alignment approach. However, our comprehensive ShortStack (Axtell, 2013) analysis made use of the integrated butter algorithm thus preventing us from overlooking any important implications due to multiple mapping sRNAs. With this, we want to emphasize how crucial the choice of mapping strategy and parameters is for alignment rate and consequential downstream analysis.

### Meiocyte and Anther sRNA Profiles Are Shaped by phasiRNA

Both meiocytes and whole anther samples had proportionally at least twice as many uniquely mapping reads (**Supplementary Figures S2A–D**), and this is due to phasiRNAs which occur in a vast abundance and stem from non-repetitive regions.

Until a very recent comprehensive study on reproductive phasiRNAs in maize (Zhai et al., 2015), only the existence of these high-copy, clustered secondary sRNAs, triggered by miR2118 and miR2275 had been reported, particularly in rice (Johnson et al., 2009; Song et al., 2012). Their function has not been elucidated yet but aspects of their biogenesis in maize (see **Figure 1**) have been comprehensively described by Zhai et al. (2015). While the 21 nt phasiRNAs peak premeiotically and are preceded by miR2118 expression from the anther epidermis, the 24 nt phasiRNAs peak during meiosis, preceded by miR2275 originating from the tapetum layer and/or meiocytes (Zhai et al., 2015). What we see here with our special technique for obtaining isolated meiocytes, is that the premeiotic 21 nt phasiRNAs still persist in the zygotene stage and, more importantly, accumulate in meiocytes vs. the whole anthers (**Figures 2A,B**). On the other hand, 24 nt phasiRNAs which were shown to peak in abundance around the time point of our zygotene sample, are detected at a lower level in meiocytes than when averaged across whole anther tissues (**Figures 2A,B**).

**FIGURE 3:**
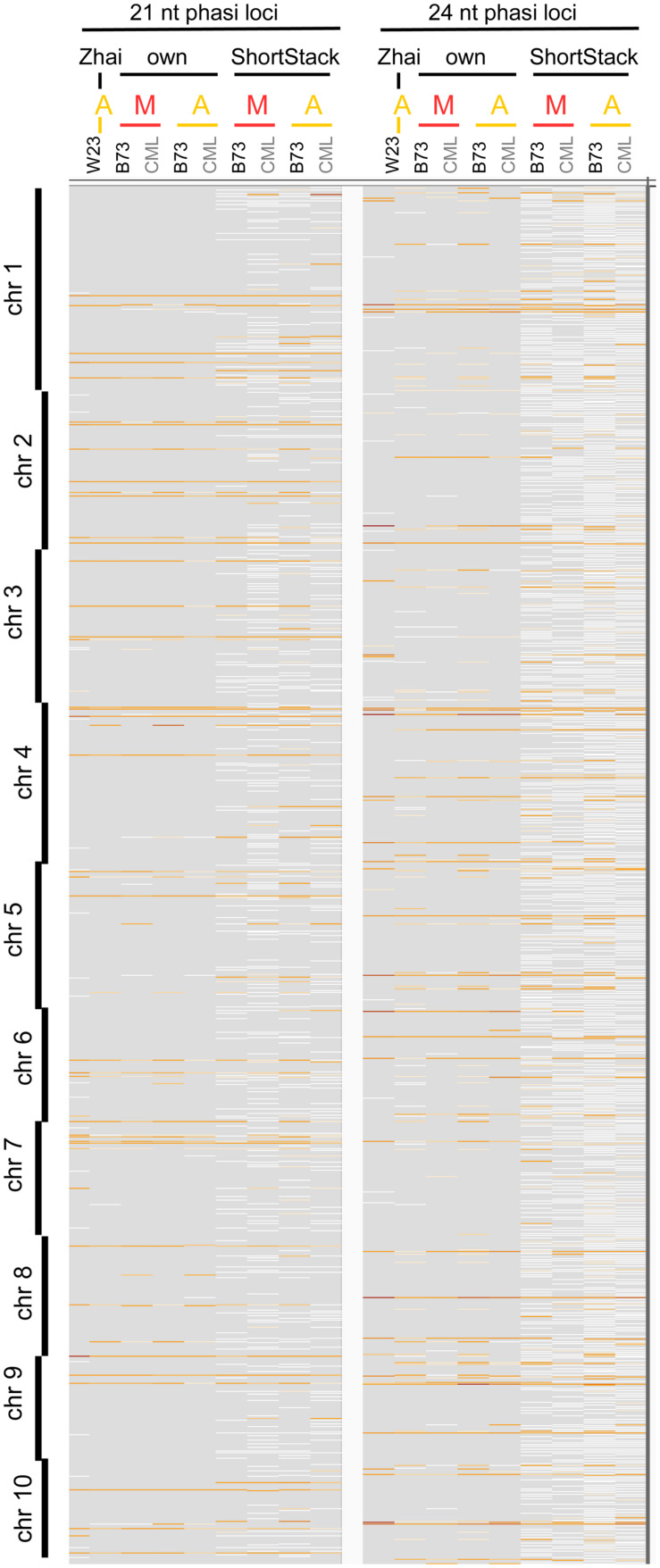
**Comparison of phasiRNA loci.** Genomic locations across all 10 chromosomes of maize are shown, from Zhai et al. (2015), our cluster calculation (defined for reads ≥2 RPM, ≤100 nt distance), and extracted from ShortStack results with a *p*-value ≤0.000000001. Background in gray, cluster loci in white (low read coverage) to dark red (high read coverage). M, meiocytes; A, anthers.

We approached the analysis of phasiRNAs or, more general, highly abundant sRNA clusters by defining clusters as having reads with less than 100 nt gaps between reads, taking only reads of at least two RPM into account. This differs from examining the read count approach we used before (**Figures 2A,B**) since the focus shifts from the total number of sRNA reads to specific genomic positions with abundant reads. As before, we detected similarity between the inbred lines, prevalence for 21 nt sRNA in meiocytes, for 24 nt sRNA in anthers, and reduced the dataset down to only a couple hundred cluster regions of interest (**Supplementary Figures S3A,B**). These clusters correspond well between anthers and meiocytes, even between inbred lines, but not with the few clusters identified in seedlings (**Supplementary Figure S3C**). The cluster lengths range from 150 nt (in 21 nt clusters in CML228 anthers) to 236 nt (in 24 nt clusters in CML228 anthers; **Supplementary Figure S4A**), and are generally specific to the sRNA species, with only the 21 nt cluster loci in B73 anthers having spikes of 24 nt sRNAs in addition (**Supplementary Figures S4B–E**). Comparing phasiRNA clusters in B73 and CML228 found by us, and in the inbred W23 in a previous publication (Zhai et al., 2015) confirms the high concordance between inbred lines, differing mainly in additional phased loci of low coverage indicated when less restrictive definitions are used via ShortStack analysis (**Figure 3**). In conclusion, we could now prove at high-resolution and on a large scale that phasiRNA are highly abundant in isolated meiocytes. Moreover, at the time-point of zygotene, 21 nt phasiRNAs prevail in meiocytes themselves while 24 nt phasiRNAs are detected at a higher level in whole anthers.

### Novel Properties of 21 and 24 nt phasiRNA Loci

**FIGURE 4:**
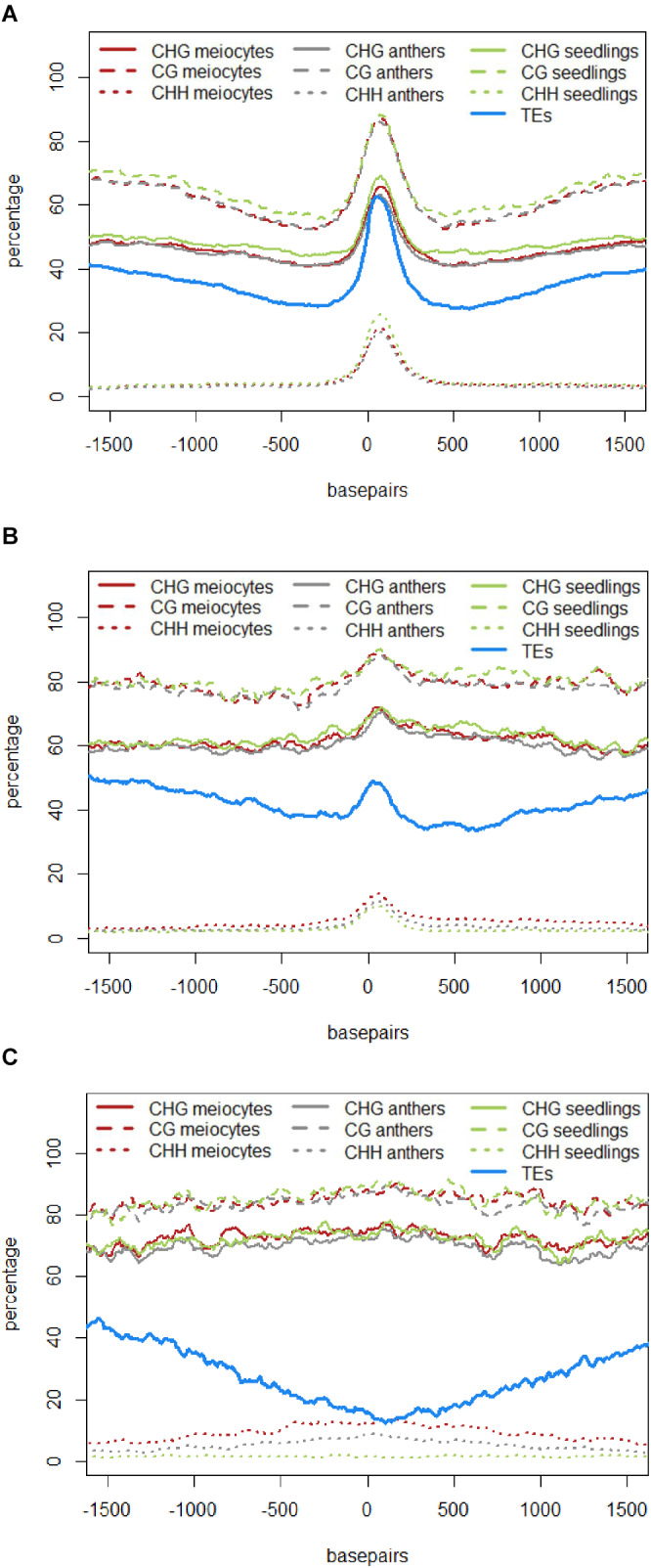
**DNA methylation at 24 nt sRNA loci.** All respective sRNA loci are aligned and plotted to start at “0,” thus including surrounding genomic regions in front of the “0” and behind the clusters, which are of variable lengths (see **Supplementary Figure S4A**). Shown is the percentage of CG, CHG, and CHH methylation averaged across all plotted sRNA loci, as well as the proportion of sRNA loci overlapping a transposable element (TE). Data from B73, graphs calculated with bedtools and plotted with R. **(A)** 24 nt sRNA loci in seedlings as detected by ShortStack. **(B)** 24 nt sRNA loci in meiocytes as detected by ShortStack. **(C)** 24 nt sRNA loci in meiocytes with reads at ≥2 RPM and ≤100 nt distance.

The role of 24 nt siRNA in RdDM (RNA-directed DNA methylation) is well established, and we confirmed this in our own data in the case of the seedling control sample. For enabling unprecedented detailed analysis of isolated meiocytes, we generated bisulfite data from those as well as from anthers and seedlings. We then calculated and plotted DNA methylation coverage in different contexts together with the proportion of loci overlapping TEs e.g., on the 24 nt sRNA loci in seedlings from the ShortStack analysis which showed the well-known reported increase of methylation in all contexts (Figure 4A). However, this trend was far less pronounced when doing a parallel analysis for the 24 nt sRNA loci in meiocytes (Figure 4B). More importantly, when we used the 24 nt sRNA loci in meiocytes defined by our criteria (reads at ≥2 RPM, with gaps between reads ≤100 nt, which results in mainly phasiRNA loci), we sampled another pool of loci which were clearly more devoid of the canonical RdDM-associated 24 nt sRNAs and had even less TE overlap than flanking regions (Figure 4C). Intriguingly, CHH methylation was substantially increased in anthers and even more so in isolated meiocytes (Figures 4B,C).

**FIGURE 5:**
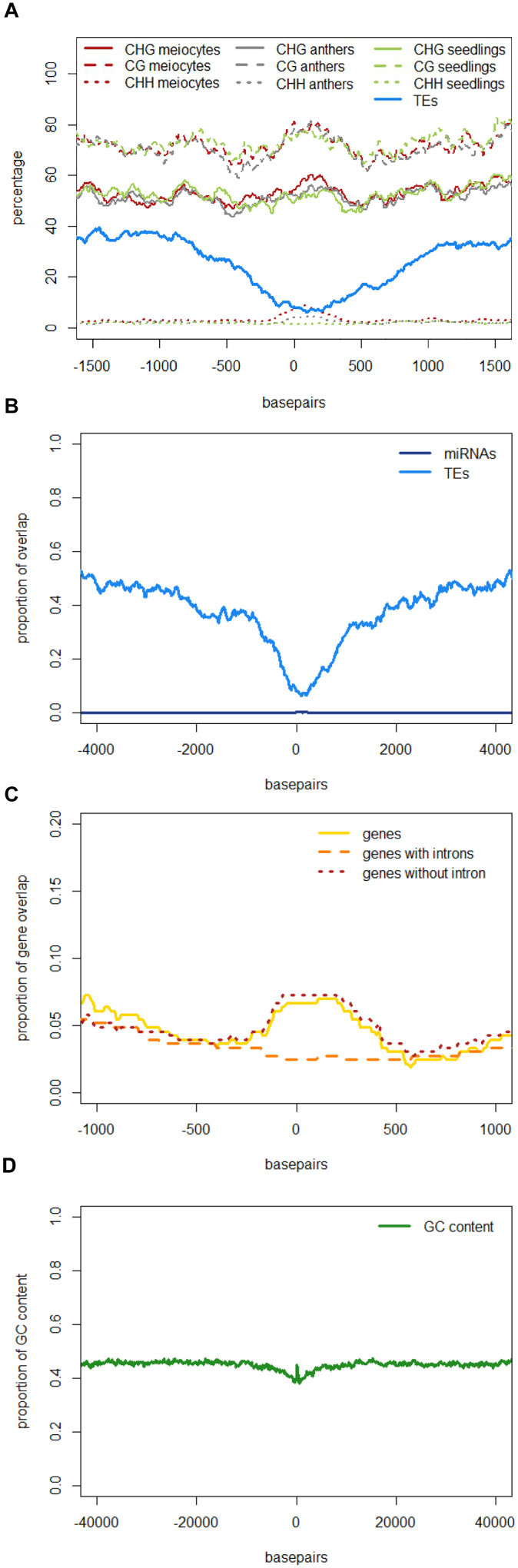
**Characteristics of 21 nt phasiRNA loci in meiocytes.** All 330 loci for 21 nt long phasiRNAs in B73 meiocytes were used to calculate averages for different genomic characteristics. PhasiRNA loci were aligned, and plotted beginning at “0,” with surrounding genomic regions on both sides. For DNA methylation, the average of the methylation percentage of the 330 loci was calculated and plotted in 100 bp windows. All other features can either be present or absent – the proportion of overlap at each of the 330 loci is checked at nucleotide resolution. Scales optimized for each feature. Data created with bedtools and plotted with R. **(A)** DNA methylation and TEs. **(B)** miRNAs and TEs. **(C)** Annotated genes, divided into genes with and without introns. **(D)** GC content of the DNA sequences.

Similar to regions with 24 nt sRNA loci, regions with 21 nt sRNA loci in meiocytes displayed CHG and CG methylation behavior without big spikes but moderate peaks and slightly higher percentages in meiocytes than in anthers and seedlings in the CHG context (Figure 5A). As for 24 nt sRNA, TE overlap was reduced at 21 nt sRNA loci, but was narrower (Figure 5A), likely due to meiocyte loci of 24 nt sRNAs having more outliers with longer cluster loci length than meiocyte loci of 21 nt sRNAs (Supplementary Figure S4A). Notably, CHH methylation showed again a distinct, very localized increase especially in isolated meiocytes when compared to seedlings and anthers, which were intermediate (Figure 5A).We characterized the 21 nt sRNA loci further regarding their overlap with genomic features, revealing a very minor co-occurrence with annotated miRNAs, the substantial dip in local TE occurrence (Figure 5B), a coverage increase with respect to annotated genes which stemmed solely from genes without introns (Figure 5C), and a peculiar pattern in their GC content, with a pronounced peak in an otherwise dip in GC content at larger scale (Figure 5D). Of these, the observation for a slight increase in annotated intronless genes might be the least relevant since they are likely lincRNAs (long intergenic non-coding RNAs) which are the precursors of the phasiRNAs; intronless genes have also been shown to have higher sRNA densities than genes with introns, with the conclusion that splicing can suppress silencing (Christie et al., 2011).

Taken together, we discovered novel characteristics of 21 and 24 nt phasiRNA loci in addition to confirming that they reside mainly in intergenic regions. PhasiRNA loci in meiocytes show higher DNA methylation in CHG, CG, and CHH context in comparison to anthers and seedlings – the highest increase is in the CHH context, and the distinction to anthers and seedlings is more pronounced in 21 nt phasiRNA loci than in 24 nt phasiRNA loci (**Figures 4C** and **5A**). In addition, both kinds of phasiRNA loci show a peculiar GC content pattern, having lower percentage of guanidines and cytosines in their vicinity than at their respective position or the global GC level (**Figure 5D**).

### Known and Novel miRNAs in Meiocytes and Anthers

Micro RNAs only constitute a small fraction of a cell’s total sRNA (**Supplementary Figures S1A,B**). However, since miRNAs are distinct from other sRNAs by virtue of their biogenesis, and their possible *trans* target gene prediction and evaluation, many have been well studied and characterized individually. MiR family members can be expressed in different tissues and have shared as well as distinct target genes. Multiple members of MIR2275 and MIR2118 are significantly up-regulated in meiocytes vs. seedlings (**Table 2**), connected with their role in production of secondary sRNAs, specific to male reproductive organs, phasiRNAs. Furthermore, MIR399b, MIR169k and o, MIR159b and k, MIR529 and MIR167g were up-regulated in meiocytes vs. seedlings (**Table 2**), all of which except MIR399 have been described as functioning in flower development or male reproductivity (Achard et al., 2004; Millar and Gubler, 2005; Schwab et al., 2005; Wu et al., 2006; Cartolano et al., 2007; Zhang et al., 2009; Jeong et al., 2011). MI399 targets a gene involved in phosphate homeostasis, and this regulatory system has later been reported as impacting flowering time in ambient temperature (Kim et al., 2011). None of the expression level of these MIR’s was significantly different between meiocytes and whole anthers, indicating importance for the whole male reproductive organ instead of for a specific meiotic process. Remarkable is that the gene expression of most of their predicted target genes does not follow the simplified dogma of down-regulation by miRNA’s, often even being more highly expressed than in seedlings (**Supplementary Table S1**). Consequently, we looked at MIR’s down-regulated in meiocytes vs. seedlings which revealed MIR168a and b, MIR169c, d, i, j, m, q, and r, MIR159f, MIR397b, MIR156d, k and l, and MIR1432 (**Table 2**).

**Table 2:** Differentially expressed miRNAs.

miRNA	Meiocytes (M)	Anthers (A)	Seedlings (S)	M vs. S	M vs. A
**Up in meiocytes (vs. seedlings)**						
MIR2275b	156716.4	180928.1	78.2	2003.3*	0.9
MIR2275c	63281.4	106643.0	38.3	1650.9*	0.6
MIR2275a	24030.8	29790.0	21.5	1117.1*	0.8
MIR2118g	7515.5	6479.2	6.6	1130.3*	1.2
MIR2118d	4987.5	4356.5	10.6	472.3*	1.1
MIR2118e	1141.1	734.9	0.4	2917.3*	1.6
MIR2118a	2945.6	3654.9	5.9	502.1*	0.8
MIR2118c	2122.0	2514.1	3.5	602.8*	0.8
MIR2118f	855.1	239.2	0.8	1093.1*	3.6
MIR2118b	6689.0	4580.4	62.2	107.6	1.5
MIR399b	514.8	602.3	3.1	164.5	0.9
MIR169o	680.6	1788.7	7.8	87.0	0.4
MIR159b	1804.5	623.3	36.8	49.1	2.9
MIR159k	566.2	429.8	11.7	48.3	1.3
MIR529	49008.2	65000.4	7484.0	6.5	0.8
MIR169k	2385.1	5074.0	168.2	14.2	0.5
MIR167g	592.0	7185.1	72.0	8.2	0.1
MIR2275d	17.2	2.9	0.8	21.9	5.9
**Down in meiocytes (vs. anthers)**						
MIR168b	411.8	3281.7	4222.3	0.098	0.125
**Down in meiocytes (vs. seedlings)**						
MIR169c	14.3	23.2	3537.0	0.004	0.617
MIR159f	0.0	1.4	531.2	0.000	0.000
MIR169j	5.7	0.7	975.9	0.006	7.892
MIR169r	0.0	0.7	340.3	0.000	0.000
MIR169i	2.9	2.9	509.3	0.006	0.986
MIR168a	486.2	2232.2	9399.4	0.052	0.218
MIR169d	0.0	2.2	224.1	0.000	0.000
MIR397b	963.7	1230.6	13721.5	0.070	0.783
MIR156k	20.0	47.1	943.4	0.021	0.425
MIR169m	5.7	8.7	473.7	0.012	0.658
MIR156d	28.6	49.3	781.5	0.037	0.580
MIR168b	411.8	3281.7	4222.3	0.098	0.125
MIR156l	5.7	13.0	248.8	0.023	0.438
MIR1432	0.0	5.1	81.0	0.000	0.000
MIR169q	0.0	4.3	72.4	0.000	0.000


*Normalized read counts of known miRNAs deposited in miRBase. Only miRNAs with a *p*-value ≤0.5 (asterisks ≤ 0.1) in the B73 samples are listed, ordered by their significance.*

**FIGURE 6:**
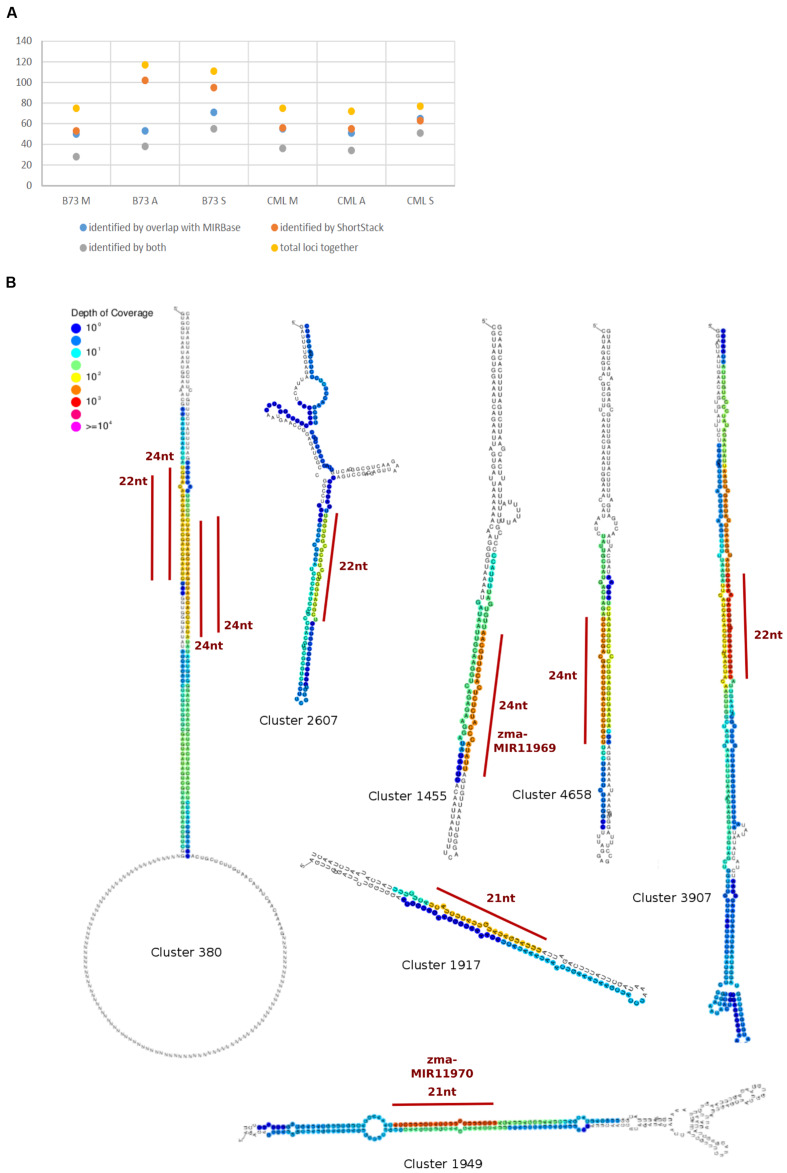
**Known and novel miRNAs.** **(A)** Comparison of miRNA identification strategies. **(B)** Hairpins derived from sRNA clusters detected in B73 meiocytes via ShortStack analysis. Locations of highest sRNA read mapping and size of putative novel miRNAs are highlighted with a red line.

To detect and analyze novel miRNAs, we supplied ShortStack with information on the genomic coordinates of known miRNA genes. We extracted all sRNA clusters that were defined as either miRNA loci by ShortStack or overlapped with annotated miRNA genes. Both strategies yielded similar amounts of loci in B73 meiocytes and in all CML228 samples, but in B73 anthers and seedlings, up to twice as many were predicted by ShortStack (**Figure 6A**). Since, we are especially interested in sRNA during meiosis, we further investigated putative novel miRNA loci in B73 meiocytes, as predicted by ShortStack. Of 24 candidate loci, 17 turned out to be novel family members of existing plant MIR families, to which they partially aligned when BLAST search was performed. Seven of the 17 even had miRBase entries for *Zea mays* miRNAs. Of the remaining seven novel miRNA loci identified by ShortStack, the most prevalent sequence was designated as the canonical miRNA (**Figure 6B**) and again cross-searched against the miRBase. The sequence extracted from Cluster 4658 had a positive hit in the plant kingdom, i.e., *Arabidopsis* miR8167. All others are promising candidates for completely novel miRNAs, although not all have the traditional length of 21 nt (two each of 21, 22, and 24 nt). The miRNAs stemming from Cluster 380 are peculiar since they occurred in pairs of slightly overlapping entities (**Figure 6B**). We followed up on the miRNA sequences from Cluster 1455 and 1949, which submitting them to the miIRBase database (Griffiths-Jones, 2006), where they are now listed under the IDs zma-MIR11696 and zma-MIR11970, respectively. These two novel miRNAs had the highest expression in meiocytes, and we predicted their target genes via the psRNATarget tool (Dai and Zhao, 2011), adding values for minimum free energy (MFE) by the TAPIR tool (Bonnet et al., 2010). mRNA-seq expression values of our previous study for these target genes illustrated again ambient trends, from lowest to highest expression in meiocytes (**Table 3**). Notably, one of the predicted target genes was RAD51C which plays an important role in both meiotic recombination and somatic DNA repair by homologous recombination. However, the predicted target site is located in an intron and might thus be a false positive or an usual fail-safe mechanism to prevent unspliced mRNA from translation, which is awaiting experimental investigation.

**Table 3:** Predicted target genes of two novel miRNAs from meiocytes.

			Gene expression (RPM)					
						
Target accession	InterPro description	Note	Meiocytes	Anthers	Seedlings	Inhibition	UPE	MFE
**For zma-MIR11969 (UUAUACCCAUCUCUCACCUUGCAA)**								
GRMZM5G868767	HCNGP-like	Putative transcription factor	62.06	42.60	21.89	Translation	19.804	-32.8
GRMZM2G060061	Glycosyl transferase, family 14		22.82	36.57	16.65	Cleavage	18.606	-37.8
GRMZM2G030139	ATPase-like, ATP-binding domain	Putative pyruvate Dehydrogenase kinase	38.05	19.56	39.68	Cleavage	20.325	-35.6
GRMZM2G041247	0	Ribonuclease H-like	0.01	0.00	0.04	Cleavage	16.515	-34.2
GRMZM2G087406	0	Ribonuclease H-like	#N/A	#N/A	#N/A	Cleavage	16.515	-34.2
GRMZM2G119261	Zinc finger, ZZ-type	Homeodomain-like	20.27	12.85	7.62	Cleavage	22.181	-33.1
GRMZM2G336583	RNA recognition motif, RNP-1	Zinc finger like	19.79	26.35	13.29	Translation	12.477	-25.4
GRMZM5G821637	Ankyrin repeat		3.22	6.05	363.23	Translation	18.004	-27.0
**For zma-MIR11970 (UGGUUUGGUUGCACGUUUGCA)**								
GRMZM2G048366	0		50.06	76.63	26.79	Cleavage	17.496	-35.3
GRMZM2G078924	Membrane-anchored ubiquitin-fold protein, HCG-1	Ubiquitin fusion protein	10.05	3.52	5.76	Cleavage	13.847	-35.3
GRMZM2G134430	Uncharacterized protein family UPF0220	Transmembrane protein	114.60	93.53	34.97	Cleavage	15.45	-32.9
GRMZM2G097106	GTPase EngC		165.27	119.39	52.91	Cleavage	20.168	-29.2
GRMZM2G123089	DNA recombination and repair protein Rad51, C-terminal	Rad51C	19.12	14.47	8.97	Cleavage	17.826	-33.4
GRMZM5G889052	Protein of unknown function DUF946		1.71	0.70	1.96	Cleavage	18.226	-33.1
GRMZM2G126197	Zinc finger, CCCH-type	Zinc finger	1.13	2.15	2.55	Cleavage	12.39	-28.7
GRMZM2G022787	Zinc finger, CCHC-type	Zinc finger	17.99	10.52	11.06	Cleavage	16.134	-31.6
GRMZM2G124143	Ribosomal protein S28e	Ribosomal protein	94.23	66.37	87.60	Cleavage	15.076	-31.4
GRMZM2G177052	Protein of unknown function DUF966		4.62	3.82	10.24	Translation	21.539	-31.2
GRMZM2G447785	Proteinase inhibitor I12, Bowman-Birk		286.86	244.96	47.03	Cleavage	15.814	-28.2
GRMZM2G304274	Glycosyl transferase, family 20		27.88	20.07	7.95	Translation	16.26	-29.9


*Target genes predicted with psRNATarget. Gene expression values from our B73 RNA-seq data. UPE, unpair energy (required minimum energy to open secondary structure around target site, values 0–100, the smaller the better). MFE, Minimum free energy (in kcal/mol, the lower the better).*

### Functional Categorization of Genes Overlapped by sRNA Loci

Other sRNAs besides miRNAs can play important roles in gene regulation or cellular function, but are often neglected due to their overwhelming abundance. We tried to mitigate this bias by approaching the analysis of all sRNA cluster loci at ≥2 RPM (calculated and characterized via the software package ShortStack) by different means. For this, we first extracted all genes overlapped by any of these loci and compared their numbers between B73 samples (**Supplementary Figure S5A**), which showed a comparable trend for CML228. We then queried for enriched biological processes in the anther and meiocyte samples via the GO annotation and analysis tool AgriGO. GO terms enriched in B73 meiocytes and anthers are centered on DNA packaging, i.e., nucleosome assembly (**Supplementary Figure S5B**). Genes identified within this category are GRMZM2G387076, GRMZM2G003306, GRMZM2G047813, GRMZM2G041381, GRMZM2G112912, GRMZM2G046841, GRMZM2G305046, GRMZM2G028955 which all encode for core histones, mainly H2A, also H2B and H3. The sRNA size distribution at these loci consistently shows the highest amount at a length of 22 nt.

A comparison between meiocyte and anther samples from both inbred lines revealed a consistent high significance of enrichment for ribosomal protein genes (81 of 5239 sRNA clusters in meiocytes, **Supplementary Figure S6**). Examples of genes encoding for ribosomal proteins overlapped by sRNA clusters are listed in **Supplementary Table S2**. This overlap enrichment can also be found in the seedling samples albeit at lower levels. Seedlings alone showed enrichment for sRNA overlap with genes for photosynthesis, and their sRNA size distribution is comparable to genes in **Supplementary Table S2**. Intriguingly, the nucleotide length of these sRNAs are usually outside the typical “functional” range of 21–24 nt. Only one of these sRNA clusters overlapping a ribosomal protein gene was identified as likely generated by a Dicer, having prevalently phased 21 nt sRNAs (**Supplementary Table S2**). Manual curation of the data overlapping ribosomal protein genes revealed one other instance of a 21 nt Dicer Call, as well as two instances of 24 nt Dicer Calls exclusively in B73 samples (**Supplementary Table S2**). While sRNA expression at both 24 nt Dicer Call locations were also detected in anther and seedling samples, none was detected in seedlings for both 21 nt Dicer Call locations. Corresponding gene expression in our mRNA-seq data shows higher gene expression of these overlapping ribosomal proteins L15 and L22/L17 in male reproductive tissues during meiosis (Dukowic-Schulze et al., 2014a).

Taken together, genes encoding core histones are enriched for 22 nt sRNAs, specifically in B73 meiocytes and anthers, indicating controlled regulation of these, while the many sRNAs overlapping ribosomal protein-encoding genes in all reproductive and vegetative control samples are of variable size, indicating general degradation.

### Comparison of sRNA Profiles from B73 and CML228

Our sRNA-seq data from B73 and CML228 showed general concordance e.g., regarding read length size distribution (**Figures 2A,B**), overlap of genomic features (**Supplementary Figures S1A** and **S2B**), read alignment to the B73 reference genome (**Supplementary Figures S2A–D**), amount and properties of clusters ≥2 RPM (**Supplementary Figures S3A**–C and **S4A**), and locations of phasi loci (**Figure 3**). Genes overlapped by sRNA loci ≥2 RPM also had common GO categories enriched between B73 and CML228, especially regarding ribosomes, but also GO categories that were distinct, particularly the enrichment for DNA packaging-related categories for B73 but not CML228 reproductive samples (**Supplementary Figure S7**). However, we have to be cautious about absolutely relying on conclusions since genes and genomic sequences present only in the CML228 genome cannot be included in any analysis because a CML228 reference genome is missing and CML228 data has to be aligned to the B73 reference genome. Many hit genes are unique to B73 (44.7%) or CML228 (20.5%; **Supplementary Figure S7A**), and the lower percentage of unique hit genes in CML228 might be due to the absence of CML228-only genes in the B73 reference genome. Still, the alignment rates for both inbreds did not differ drastically (∼5–20%, **Supplementary Figure S2**), indicating conservation of most phasiRNA loci and many TEs whose genomic rearrangement is likely but does not influence mapping success.

Looking at all sRNA loci at ≥2 RPM (not just phasiRNA loci or the loci that overlapped genes) highlights that the sRNA data especially from the reproductive samples correlate well between the inbred lines but that anthers and meiocytes from the same inbred have a higher correlation with each other than their counterparts from the other inbred line (**Supplementary Figure S7B**). Surprisingly, sRNA loci that showed ≥10-fold difference between B73 and CML228 meiocytes have similar profiles across chromosomes (e.g., with many of these differentially expressed loci in the first 15% of chromosome 6), with rather pronounced local changes (**Supplementary Figure S7C**). MA plots which depict the expression value intensity differences as a scatterplot show more differential expression of loci in meiocytes than in anthers between the two accessions (**Supplementary Figures S7D,E**), possibly indicating a high conservation of the anther development process in contrast to climate or other adaptation of the meiotic process.

## Discussion

Here, we analyzed next-generation sequencing data of small RNAs and implications on the DNA methylome of isolated male meiocytes from *Zea mays*. Since generating the biological material is the bottleneck in the experiment setup, we pooled multiple individuals in each sample tissue type and pursued the analysis with one B73 and one CML228 sample each, hypothesizing that we could thus confirm tendencies observed from the B73 samples, and in addition gain added value by finding clues on biologically significant differences.

### Implications from sRNA Read Alignment, Analysis, and Size Distribution

The alignment strategy influences all downstream analyses. In order to mitigate any possible detrimental impact due to the selected alignment method, we used different algorithms and parameters, i.e., conventional GSNAP (Wu and Nacu, 2010) allowing up to five genomic locations for a read, GSNAP with up to 85 genomic locations, and the bowtie-based butter aligner for mapping reads with up to 1000 genomic locations if they can be assigned due to density (Johnson et al., 2016). Analysis focused on uniquely aligning reads can be done on any of these alignments, while the high amount of TEs in the maize genome calls for the less restrictive strategies, especially when TE silencing and heterochromatin regulation are of prime interest. Among all algorithms used, density-based mapping seems to be a very valuable approach, making use of the most sRNA reads (**Table 1**).

Maybe at first counterintuitive, we disregarded many reads for most downstream analyses after we had carefully applied different alignment strategies to maximize true read mapping. However, limiting analysis to regions of at least two RPM eliminated background noise throughout the genome which stem from e.g., degrading mRNAs, and facilitated downstream analysis. Substantial mRNA degradation of biological significance can still be detected, as demonstrated by abundant sRNA reads mapping to ribosomal protein genes (**Supplementary Table S2**). Those instances, regardless whether sRNA size distribution points to specific regulation through a dicer-dependent sRNA species, or to general degradation with sRNAs of diverse lengths, are in agreement with cytological observations of ribosome depletion during meiotic prophase (Mackenzie et al., 1967; Dickinson and Heslop-Harrison, 1977) and with decrease of ribosome gene expression levels during anther development (Crismani et al., 2006).

### miRNAs and sRNA Pathway Components

In spite of the decrease in ribosomes, translation is not completely abolished during meiosis, and can be regulated by miRNAs, the most prominent sRNA species. Most miRNAs have target genes, and spatially down-regulate or dampen their expression post-transcriptionally (Jofuku et al., 1994; Chen, 2004; Cartolano et al., 2007). However, the simplistic view of miRNAs as mere down-regulators has to be taken with caution since (i) miRNAs were proposed to rather have a role in buffering noise at intermediate expressed genes (Hornstein and Shomron, 2006), (ii) miRNAs can down-regulate DNA methylation, consequently increasing gene expression (reviewed in: Peschansky and Wahlestedt, 2014), and (iii) miRNAs can also up-regulate translation (Vasudevan et al., 2007). In our data, mRNA levels of predicted target genes of known and novel miRNAs up-regulated in meiocytes did often show higher expression in the reproductive samples, possibly due to one of the aforementioned mechanisms. In addition, distinct family members of MIR169 and MIR159 were up- or down-regulated in meiocytes, pointing to an intricate fine-tuning of them and their target gene expression. Adding to the complexity by regulating the sRNA targeting machinery itself, MIR168b which regulates *Ago1* homeostasis through a negative feedback loop during development (Vaucheret et al., 2004, 2006; Ding et al., 2009) is the only MIR differentially expressed between meiocytes and anthers (**Table 2**).

Further interesting sRNA pathway component genes regarding meiosis are (i) *Dcl3b* which is practically absent from seedlings (**Figure 1A**) and likely involved in 24 nt hc-siRNA biogenesis (Xie et al., 2004), (ii) *Rdr1* and *Rdr2* which both have their highest expression value in meiocytes, and (iii) *Ago’s 2, 5a, and 18a/b/c* (**Figure 1A**). *Ago5c* and *Ago18b* were suggested to bind phasiRNAs of 21 and 24 nt length, respectively (Zhai et al., 2015). Maize *Ago5c* is the closest homolog of rice *MEL1* which binds 21 nt phasiRNAs, regulates chromosome condensation in male and female meiosis, and leads to meiotic arrest when mutated (Nonomura et al., 2007).

While none of the maize *Ago1* homologs seems to be up-regulated in meiocytes vs. anthers, *Ago1d* is preferentially expressed in both reproductive samples vs. seedlings with the level in anthers approximately twice as high as in meiocytes (**Figure 1B**). An ARGONAUTE of confirmed importance for meiosis is Ago104 which has been reported earlier as a non-cell-autonomously acting key component for female meiosis, causing apomixis, i.e., unreduced viable gametes, when mutated (Singh et al., 2011). *Ago104* was also shown to be needed for heterochromatic CHG and CHH methylation, and regulating chromosome condensation and disjunction in male meiosis (Singh et al., 2011). Similar to *Ago104* in maize, its homolog in *Arabidopsis*, *AtAGO9*, is also involved in RdDM, and mutants exhibit many chromosome interlocks starting in pachytene of prophase I (Oliver et al., 2014). Of note, *Ago9* is primarily expressed in the nucellus, from where the DNA methylation landscape of female spore mother cells is likely influenced via mobile sRNAs (reviewed in: Baroux and Autran, 2015). *Ago104* is also a great example for an sRNA pathway component acting close to meiotic cells by producing mobile signals which then impact meiotic cells (Singh et al., 2011).

### Mobility of sRNAs and Morpho-gradient Formation

Interestingly, the mobility of sRNA seems to differ depending on the RNA species and/or their biogenesis. As an example, tasiRNAs can act on a wider range than miRNAs (de Felippes et al., 2011). Cell-to-cell movement of small RNAs as in the case of tasiRNA-ARFs (low abundant *Arabidopsis* tasiRNAs which target ARF3, an auxin response factor) leads to a concentration gradient in the leaf (Chitwood et al., 2009), and is also important for the shoot apical meristem (SAM) and for anther development in rice (Nagasaki et al., 2007; Toriba et al., 2010). These tasiRNA examples strengthen a proposed function for phasiRNAs in anther development since phasiRNAs are also mobile and generate a gradient across the anther, but are far more abundant (Zhai et al., 2015 and this study). Though this is a possible scenario, so far, the exact function of phasiRNA is unknown (Johnson et al., 2009; Ding et al., 2012; Song et al., 2012). Besides forming morpho-gradients due to sRNA-mediated target gene regulation, mobile sRNAs also influence DNA by supporting heterochromatin formation and possibly facilitating chromosome dynamics and condensation during meiosis with it (Singh et al., 2011; Oliver et al., 2014). In these cases, meiotic cells might thus be saved from using resources on producing necessary sRNAs, instead getting them supplied by outer layers, as is the case for nutrients supplied from tapetal cells, and was also suggested for phasiRNAs by Zhai et al. (2015).

### Putative Functions of phasiRNAs

What the mammalian (pachytene) piRNAs and plant phasiRNAs have in common are their origin from intergenic, non-repetitive, unannotated regions. This is particularly interesting considering the functional implication: Abundant siRNAs are often primarily seen as targeting repetitive regions for mRNA degradation or DNA methylation, and since they also need a genomic sequence where they stem from, this should already lead to at least two hits in the genome. This means that the uniquely mapping sRNAs (**Supplementary Figure S2**) are rather targeting only their own sequence context. In animals, functional sRNAs often have only a core complementary sequence for their target, but plant sRNA-target complementarity is frequently a perfect one. In agreement with that, no target genes could be predicted for plant phasiRNAs (Song et al., 2012; Zhai et al., 2015), implying a function different from miRNAs, tasiRNAs and hc-siRNAs, and arguing against a function of phasiRNAs in building a morpho-gradient.

**FIGURE 7:**
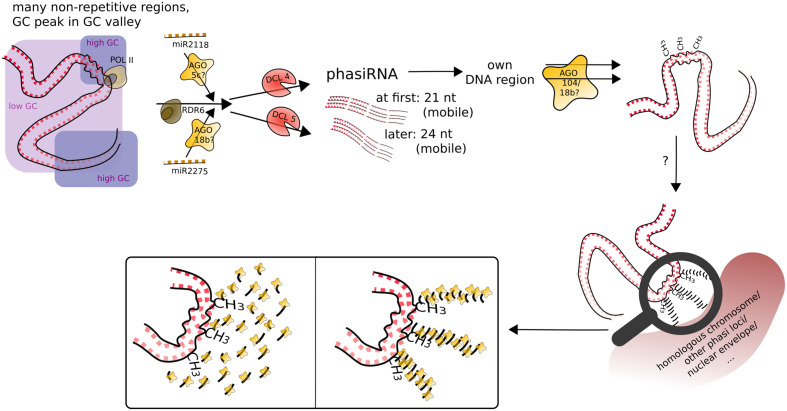
**Speculative model for phasiRNA function in meiotic chromosome dynamics.** PhasiRNA direct DNA methylation, most obviously in CHH context, to their loci of origin (cis), possibly by AGO104. In addition, phasiRNA could act as a hallmark or guide for chromatin remodeling factors (not depicted), in addition/or alternatively tethering the chromatin to another nuclear structure. Framed insets of the magnifying glass display possible conformations of accumulating phasiRNAs guided by ARGONAUTE.

What was found for some tasiRNAs, was that they can mediate DNA methylation at their loci of origin albeit without resulting in suppression of expression (Wu et al., 2012). What we can now add to the conundrum of the function of phasiRNAs is that they likely also mediate DNA methylation at their loci of origin, most obviously CHH methylation, and apparently target it to meiocytes, with whole anthers showing CHH methylation at a lesser extent (**Figures 4C** and **5A**). Good ARGONAUTE candidates for mediating this non-canonical RdDM are *Ago104* which functions in CHH and CHG methylation on heterochromatin and is important for meiosis (Singh et al., 2011), or the yet uncharacterized yet highly abundantly expressed Ago18b (**Figure 7**).

The discovery of localized, meiocyte-specific CHH methylation at phasiRNA loci from both 21 and 24 nt phasiRNAs, together with (i) the high abundance of phasiRNAs, (ii) the absent suppression at phasiRNA loci, (iii) the peculiar GC content pattern around phasiRNA loci (**Figure 5D**), and (iv) indications from the literature as described in the following, lead us to suggest a function of phasiRNA in chromatin remodeling during meiosis, possibly assisting chromosome dynamics during pairing, synapsis and/or recombination. There is substantial reorganization of chromatin during male meiosis (reviewed in: Zhou and Pawlowski, 2014), and the RNAi machinery is required to maintain Polycomb group-dependent physical long-distance interactions, clustering of telomeres and higher-order insulator complex formation (Hall et al., 2003; Grimaud et al., 2006; Lei and Corces, 2006). Our own finding of a GC-rich peak in a larger-scale GC-poor valley around the phasiRNA loci implies specific chromatin properties around those loci, lower intrinsic energy (and thus easier disintegration of the double strand helix). The DNA methylation at the GC peak adds another likely DNA rearrangement, from the usual B-form of the DNA helix to the unusual Z-form.

Although at that point not well supported, we imagine that phasiRNAs play a role similar to *Arabidopsis* diRNA (double-strand break-induced RNA) which was proposed to act as a guide for repair proteins (Wei et al., 2012). Similarly, phasiRNAs could (i) guide chromatin remodeling factors to their origin loci, or (ii) accumulate in their origin loci’s vicinity to act as a hallmark or support in restructuring chromatin or tethering it to a certain location in the nucleus or to the homologous chromosome. In the case of phasiRNA stacks, this could even be envisioned as a molecular zipper (**Figure 7**). Although 21 and 24 nt phasiRNA have different timing and spatial initialization in anthers (Zhai et al., 2015), we envision them as acting in the same way, but in two waves. This could also explain, why we detect higher DNA methylation in CG and CHG context for 21 nt phasi loci than for 24 nt phasi loci at the stage of zygotene, when 24 nt phasi only start to peak in whole anthers and might not have reached their final destination yet. Although novel to male meiosis, substantial chromatin remodeling during meiosis, the importance of non-cell-autonomous mobile small RNA signals in chromatin organization, and two waves of chromatin dynamic changes (one premeiotic and one meiotic) have already been highlighted in plant female meiosis (reviewed in: Baroux and Autran, 2015). Furthermore, H2AZ loading in male meiosis was suggested to play a role providing an instructive template for crossovers (Choi et al., 2013), and histone hyperacetylation as well as DNA methylation mutations can lead to altered recombination (Perrella et al., 2010; Mirouze et al., 2012; Yelina et al., 2012).

## Conclusion

We uncovered two novel meiotic miRNAs and indications for a putative function for phasiRNAs in DNA methylation. Our speculative model for phasiRNAs acting in chromosome dynamics, if confirmed by experimental approaches, might lead to a re-consideration of the current classification of phasiRNAs as canonical small interfering RNAs. Here, we only laid out the foundation for the intriguing though not yet well supported possibility for a phasiRNA function in DNA methylation and/or chromosome dynamics. We hope that our hypotheses can be tested in the future by detailed characterizations including computational modeling of DNA at phasiRNA loci with abundant phasiRNAs, study of DNA methylation and meiotic chromosome behavior in sRNA pathway mutants, and by DNA and RNA FISH (fluorescence *in situ* hybridization) and/or Hi-C sequencing as described in Kaplan and Dekker (2013) following the three-dimensional localization of phasiRNA loci throughout meiosis.

## Author Contributions

SD-S performed lab experiments, AS, TR, and SD-S conducted the data analysis. SD-S wrote the manuscript. CC, JM, WP, and SK designed the original research. All authors edited the manuscript and approved the final version.

## Conflict of Interest Statement

The authors declare that the research was conducted in the absence of any commercial or financial relationships that could be construed as a potential conflict of interest.

## Footnotes

1 http://bar.utoronto.ca/efp_maize
