# Corrigendum: Novel Meiotic miRNAs and Indications for a Role of PhasiRNAs in Meiosis

**DOI:** 10.3389/fpls.2020.00653

**Published:** 2020-06-04

**Authors:** Stefanie Dukowic-Schulze, Anitha Sundararajan, Thiruvarangan Ramaraj, Shahryar Kianian, Wojciech P. Pawlowski, Joann Mudge, Changbin Chen

**Affiliations:** ^1^Department of Horticultural Science, University of Minnesota, St. Paul, MN, United States; ^2^National Center for Genome Resources, Santa Fe, NM, United States; ^3^Cereal Disease Laboratory, United States Department of Agriculture - Agricultural Research Service, St. Paul, MN, United States; ^4^Section of Plant Biology, School of Integrative Plant Science, Cornell University, Ithaca, NY, United States

**Keywords:** meiosis, meiocytes, small RNA, phasiRNA, DNA methylation, maize, sRNA-seq, bisulfite sequencing

In the original article, there was a mistake in Figures 4A–C and Figure 5A as published. The figures depicting DNA methylation underrepresented the true DNA methylation in our samples and in maize in general. The underlying computation made use of the average DNA methylation in 100 bp tiles. However, there were tiles that either had no DNA methylation sites or had no read calls at existing DNA methylation sites. Instead of disregarding these instances, tiles with non-existent values were used in the original computation as the value “0%.” We now removed any instances of tiles with unknown or absent DNA methylation information. Due to the different likelihoods of these instances in different contexts, substantial differences in the calculated DNA methylation percentage occurred in the CG context, less in the CHG and almost none in the CHH context (H = A, T, or C). The higher DNA methylation percentages in the corrected graphs agree with other maize DNA methylation data. The correction did not change the previously described qualitative outcomes. The corrected Figures 4A–C and Figure 5A appear below. The following sections have been updated correspondingly. In addition, since two of the authors changed institutions by now, their new affiliations are listed in the author section of this corrigendum.

**The Methods section, subsection sRNA Analysis:**

“SAMTools (Li et al., 2009) was used via the Unix command line to extract data from BAM alignment files for the production of Excel graphs for size distribution, read mapping, and genomic feature overlap. Aligned reads were visualized with IGV (Integrative Genomics Viewer, Broad Institute; Robinson et al., 2011), with improved calculation and displaying facilitated by created TDF files. Exaggerated background read reduction for diverse downstream analyses, including phasiRNA loci determination, was achieved by removing reads from any loci with less than two RPM (reads per million). Coverage plots and correlation heat map were computed using BEDTOOLS (Quinlan, 2014) and graphed using the R Statistical Program. Importantly, instances with no methylation information need to be ignored, and not treated as 0%. Rows with “.” were thus removed by “grep” after “bedtools map,” before “bedtools groupby.” Coverage plots are used to average e.g., DNA methylation percentage or the proportion of a feature presence over multiple loci. For example, if 100 loci of interest are analyzed for their overlap with annotated genes, and 80 of them do overlap, the coverage plot y-value at the start or mid of the loci is 80%; however, since not all loci or hit genes have the same length, the percentage decreases when proceeding on the y-axis. For effects of sRNAs in *trans*, differential expression of miRNAs was tackled by generating read counts for miRBase (Griffiths-Jones, 2006) entries for maize with our initial GSNAP alignment, and also by running ShortStack analysis with a flagfile which included known miRNA gene loci. BLASTN (task blastn-short) algorithm from the NCBI BLAST+ suite (Camacho et al., 2009) was run via Unix Command Line to check whole sRNA cluster regions annotated by ShortStack as miRNA against miRNAs listed in the miRBase database. The resulting short miRNA sequences were checked directly online against miRBase with SSEARCH parameters. Target gene prediction for putative miRNAs was performed with psRNATarget (Dai and Zhao, 2011). For the effect of sRNAs in *cis*, all genes overlapping sRNA clusters identified by ShortStack were analyzed for overlaps between samples via BioVenn (Hulsen et al., 2008) and Venny (Oliveros, 2007), and subjected to GO (Gene Ontology) annotation via AgriGO (Du et al., 2010). Examination of differentially expressed sRNA loci was done using ShortStack in count mode (Axtell, 2013), and the Bioconductor DEseq package for R (Anders and Huber, 2010).”

**The Results section, subsection Novel Properties of 21 and 24 nt phasiRNA Loci, paragraphs 1 and 2:**

“The role of 24 nt siRNA in RdDM (RNA-directed DNA methylation) is well established, and we confirmed this in our own data in the case of the seedling control sample. For enabling unprecedented detailed analysis of isolated meiocytes, we generated bisulfite data from those as well as from anthers and seedlings. We then calculated and plotted DNA methylation coverage in different contexts together with the proportion of loci overlapping TEs e.g., on the 24 nt sRNA loci in seedlings from the ShortStack analysis which showed the well-known reported increase of methylation in all contexts (Figure 4A). However, this trend was far less pronounced when doing a parallel analysis for the 24 nt sRNA loci in meiocytes (Figure 4B). More importantly, when we used the 24 nt sRNA loci in meiocytes defined by our criteria (reads at ≥ 2 RPM, with gaps between reads ≤ 100 nt, which results in mainly phasiRNA loci), we sampled another pool of loci which were clearly more devoid of the canonical RdDM-associated 24 nt sRNAs and had even less TE overlap than flanking regions (Figure 4C). Intriguingly, CHH methylation was substantially increased in anthers and even more so in isolated meiocytes (Figure 4B,C).

**Figure 4:**
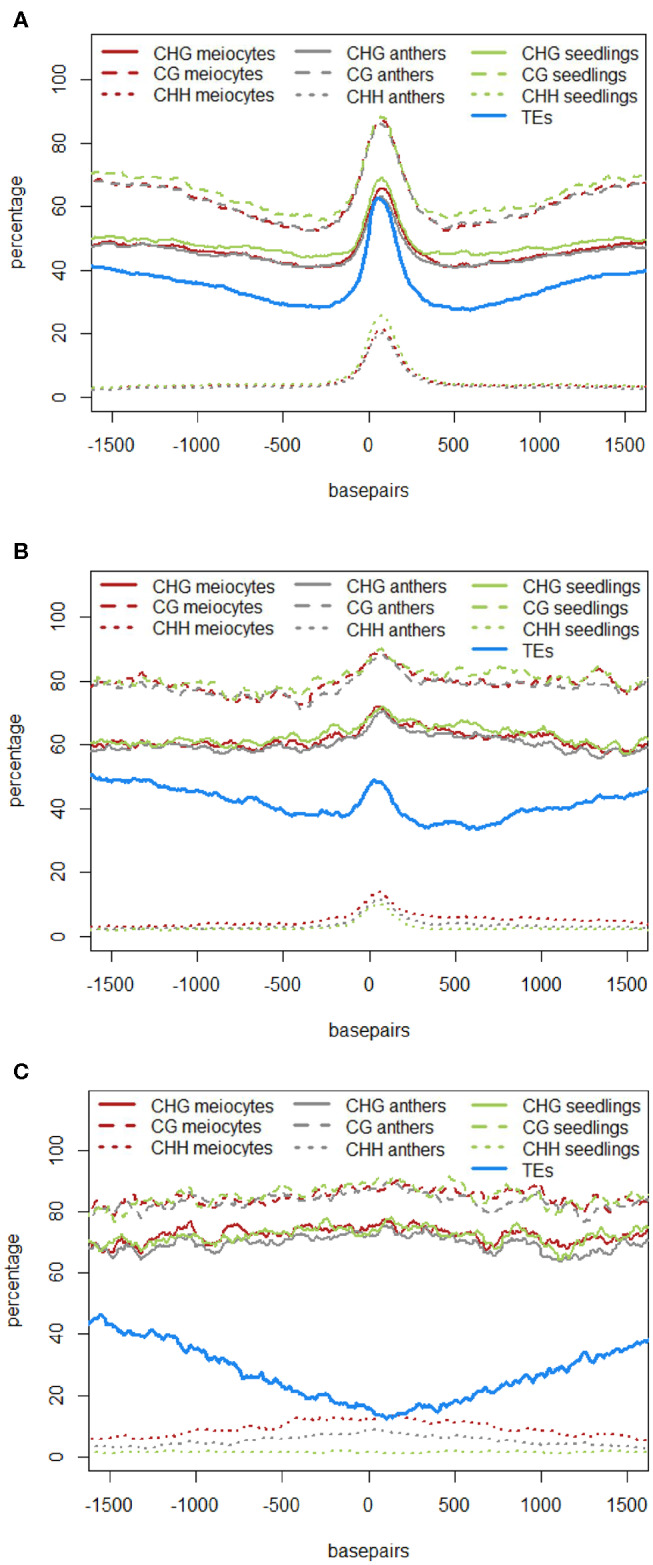


Similar to regions with 24 nt sRNA loci, regions with 21 nt sRNA loci in meiocytes displayed CHG and CG methylation behavior without big spikes but moderate peaks and slightly higher percentages in meiocytes than in anthers and seedlings in the CHG context (Figure 5A). As for 24 nt sRNA, TE overlap was reduced at 21 nt sRNA loci, but was narrower (Figure 5A), likely due to meiocyte loci of 24 nt sRNAs having more outliers with longer cluster loci length than meiocyte loci of 21 nt sRNAs (**Supplementary Figure S4A**). Notably, CHH methylation showed again a distinct, very localized increase especially in isolated meiocytes when compared to seedlings and anthers, which were intermediate (Figure 5A). We characterized the 21 nt sRNA loci further regarding their overlap with genomic features, revealing a very minor co-occurrence with annotated miRNAs, the substantial dip in local TE occurrence (Figure 5B), a coverage increase with respect to annotated genes which stemmed solely from genes without introns (Figure 5C), and a peculiar pattern in their GC content, with a pronounced peak in an otherwise dip in GC content at larger scale (Figure 5D). Of these, the observation for a slight increase in annotated intronless genes might be the least relevant since they are likely lincRNAs (long intergenic non-coding RNAs) which are the precursors of the phasiRNAs; intronless genes have also been shown to have higher sRNA densities than genes with introns, with the conclusion that splicing can suppress silencing (Christie et al., 2011).”

**Figure 5:**
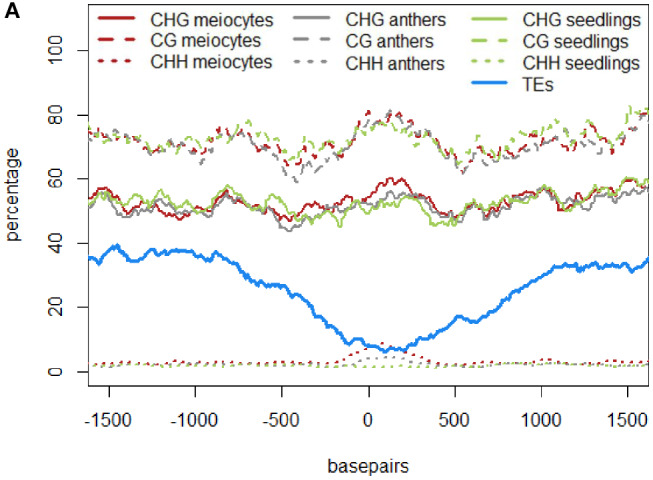


The authors apologize for this error and state that this does not change the scientific conclusions of the article in any way. The original article has been updated.
