# Supplementary material for: Novel Meiotic miRNAs and Indications for a Role of PhasiRNAs in Meiosis
Source: Front Plant Sci. 2016 Jun 2;7:762. doi: 10.3389/fpls.2016.00762 (PMC4889585; doi:10.3389/fpls.2016.00762)
Supplement: Supplementary file 1 [file Table_1.PDF]

Supplementary Table S1. Expression level of putative target genes of miRNAs up-regulated in meiocytes

Normalized read counts of putative target genes, as suggested by Zhang et al. (2009). Color coding for expression level with green = low, yellow = mid, and red = high expression. M = meiocytes, A = anthers, S = seedlings. Data from our published B73 RNA-seq. #N/A = without unique reads.

Gene ID	Interpro description	M	A	S
**MIR159b/k (17 targets)**				
GRMZM2G027100	unknown	0.0	0.5	0.7
GRMZM2G416652	Homeodomain-like	8.5	5.1	1.8
GRMZM2G167088	MYB domain transcription factor; Homeodomain-like; SANT, DNA-binding	1.2	0.0	0.0
GRMZM2G423833	MYB domain transcription factor; Homeodomain-like; SANT, DNA-binding	2319.0	1218.5	136.3
GRMZM2G061972	MYB domain transcription factor; Homeodomain-like; SANT, DNA-binding	#N/A	#N/A	#N/A
AC204352.3_FG012	**Cell division**	74.4	84.8	0.0
AC209015.3_FG004	unknown	0.0	0.0	0.0
GRMZM2G113073	PAK-box/P21-Rho-binding ;Antifreeze protein, type I; Rho GTPase activation protein; RhoGAP	696.9	823.1	2743.8
GRMZM2G093789	MYB domain transcription factor; SANT, DNA-binding; Homeodomain-like; Riboflavin kinase	1742.6	1233.7	438.9
GRMZM2G070523	MYB domain transcription factor; Small proline-rich; SANT, DNA-binding; Homeodomain-like	26.9	84.7	2.3
AC217264.3_FG005	unknown	184.0	790.6	15.6
GRMZM2G139688	MYB domain transcription factor; Homeodomain-like; SANT, DNA-binding	2153.0	3066.4	293.6
GRMZM2G386944	unknown	442.1	710.7	1.4
GRMZM2G085550	unknown	638.4	3035.1	605.4
GRMZM2G451605	unknown	656.0	1000.7	1.4
GRMZM2G075064	MYB domain transcription factor; Homeodomain-like; SANT, DNA-binding	0.0	0.5	0.0
GRMZM2G038195	Metallophosphoesterase; Serine/threonine-specific protein phosphatase and bis(5-nucleosyl)-tetraphosphatase	1449.2	1442.8	1063.6
**MIR399b (6 targets)**				
GRMZM2G165734	unknown	0.3	0.0	0.0
GRMZM2G070591	unknown	23.7	66.7	0.0
GRMZM2G081812	Zinc finger, C2H2-like; Zinc finger, LIM-type	2567.0	2045.9	2295.4
GRMZM2G070087	Major facilitator superfamily MFS-1; general substrate transporter; Phosphate permease	356.3	634.1	49.9
GRMZM2G075870	Major facilitator superfamily MFS-1; general substrate transporter; Phosphate permease	181.3	627.4	1.4

GRMZM2G112377	Antifreeze protein, type I; Major facilitator superfamily MFS-1, general substrate transporter; Phosphate permease	233.7	268.4	35.2
**MIR169i/j/k (9 targets)**				
GRMZM2G078124	Molluscan rhodopsin C-terminal tail	1660.6	914.8	427.7
GRMZM2G033245	CCAAT-binding transcription factor, subunit B	#N/A	#N/A	#N/A
GRMZM2G091964	CCAAT-binding transcription factor, subunit B; CCAAT-binding factor, conserved site	750.1	600.8	500.3
GRMZM2G000686	CCAAT-binding transcription factor, subunit B	833.1	621.6	796.4
GRMZM2G040349	CCAAT-binding transcription factor, subunit B	258.0	378.5	384.3
GRMZM2G083670	MYB domain transcription factor; Streptococcal non-M secreted SibA; SANT, DNA-binding	#N/A	#N/A	#N/A
GRMZM2G008250	unknown	40.9	38.7	25.9
GRMZM2G165488	CCAAT-binding transcription factor, subunit B	11.6	26.1	166.2
GRMZM2G038303	CCAAT-binding transcription factor, subunit B	67.4	91.3	242.0
**MIR169o (5 targets)**				
GRMZM2G165488	CCAAT-binding transcription factor, subunit B	11.6	26.1	166.2
GRMZM2G008250	unknown	40.9	38.7	25.9
GRMZM2G091964	CCAAT-binding transcription factor, subunit B	750.1	600.8	500.3
GRMZM2G078124	Molluscan rhodopsin C-terminal tail	1660.6	914.8	427.7
GRMZM2G033245	CCAAT-binding transcription factor, subunit B	#N/A	#N/A	#N/A
