# Supplementary material for: Novel Meiotic miRNAs and Indications for a Role of PhasiRNAs in Meiosis
Source: Front Plant Sci. 2016 Jun 2;7:762. doi: 10.3389/fpls.2016.00762 (PMC4889585; doi:10.3389/fpls.2016.00762)
Supplement: Supplementary file 2 [file Table_2.PDF]

Supplementary Table S2. Characteristics of sRNA loci overlapping ribosomal protein genes

sRNA read distribution at sRNA clusters overlapping ribosomal protein genes. “Strand” refers to Watson/+ strand (“1”) or Crick/- (“0”) strand, decimal numbers state the ratio between the strands of origin. Table from analysis with ShortStack. Listed genes were overlapped by sRNAs from meiocytes and anthers in both inbred lines, values from B73 meiocytes. *Additional ribosomal protein genes found only in B73 reproductive samples.

overlapping gene ID	description	Size of sRNA cluster	Strand	Total	Uniques	Dicer Call	Phase pval	nucleotide length 20	21	22	23	24	shorter (<20)	longer (>24)
GRMZM2G090422	Ribosomal protein L13	79	1	4151	4055	N	NA	656	170	34	73	657	294	2267
GRMZM2G072315	Ribosomal protein L13	344	0	6262	4823	N	NA	86	370	232	253	92	1114	4115
GRMZM2G150058	Ribosomal protein L14b/ L23e	607	1	2911	1723	N	NA	37	14	33	27	10	2261	529
GRMZM2G171181	Ribosomal protein L14b/ L23e	416	1	266	77	N	NA	0	0	2	0	1	162	101
		231	1	374	351	N	NA	0	0	0	2	4	92	276
		601	1	8180	1541	N	NA	14	5	18	6	3	7671	463
GRMZM2G322413	Ribosomal protein L18e	37	1	417	413	N	NA	2	8	25	20	28	62	272
GRMZM2G104025	Ribosomal protein L18e	583	1	2008	1710	N	NA	61	83	131	82	89	309	1253
GRMZM2G163769	Ribosomal protein L22/L17	355	0.151	9668	9662	21	1.31E-08	371	9146	23	10	34	84	0
GRMZM2G088060	Ribosomal protein L28e	37	0	632	0	N	NA	23	69	7	5	0	50	478
GRMZM2G050460	Ribosomal protein L30e	491	0	37579	4056	N	NA	556	367	1176	1944	2892	4253	26391
GRMZM2G123969	Ribosomal protein L30e	38	1	5020	0	N	NA	26	5	6	24	1	158	4800
GRMZM2G044137	Ribosomal protein L30e	366	1	5563	0	N	NA	27	17	7	30	5	173	5304
GRMZM2G027728	Ribosomal protein L30e	29	1	77	41	N	NA	0	2	2	0	13	36	24
GRMZM2G158568	Ribosomal protein L31e	287	0.239	213	189	N	NA	9	26	27	15	47	33	56
		137	0	871	816	N	NA	3	0	7	6	6	154	695
		156	0	1103	1087	N	NA	3	17	13	14	61	26	969
		77	0	1761	625	N	NA	7	2	3	5	19	27	1698
GRMZM5G852185	Ribosomal protein S15*	65	0	75	65	21	NA	5	57	3	0	2	7	1
GRMZM2G135727	Ribosomal protein L3*	83	0.985	137	137	24	NA	0	4	2	9	118	1	3
GRMZM2G109677	Ribosomal protein L3*	128	0.224	125	125	24	0.97475	0	7	1	13	96	4	4
