# Supplementary material for: Novel Meiotic miRNAs and Indications for a Role of PhasiRNAs in Meiosis
Source: Front Plant Sci. 2016 Jun 2;7:762. doi: 10.3389/fpls.2016.00762 (PMC4889585; doi:10.3389/fpls.2016.00762)
Supplement: Supplementary file 3 [file Image_1.PDF]

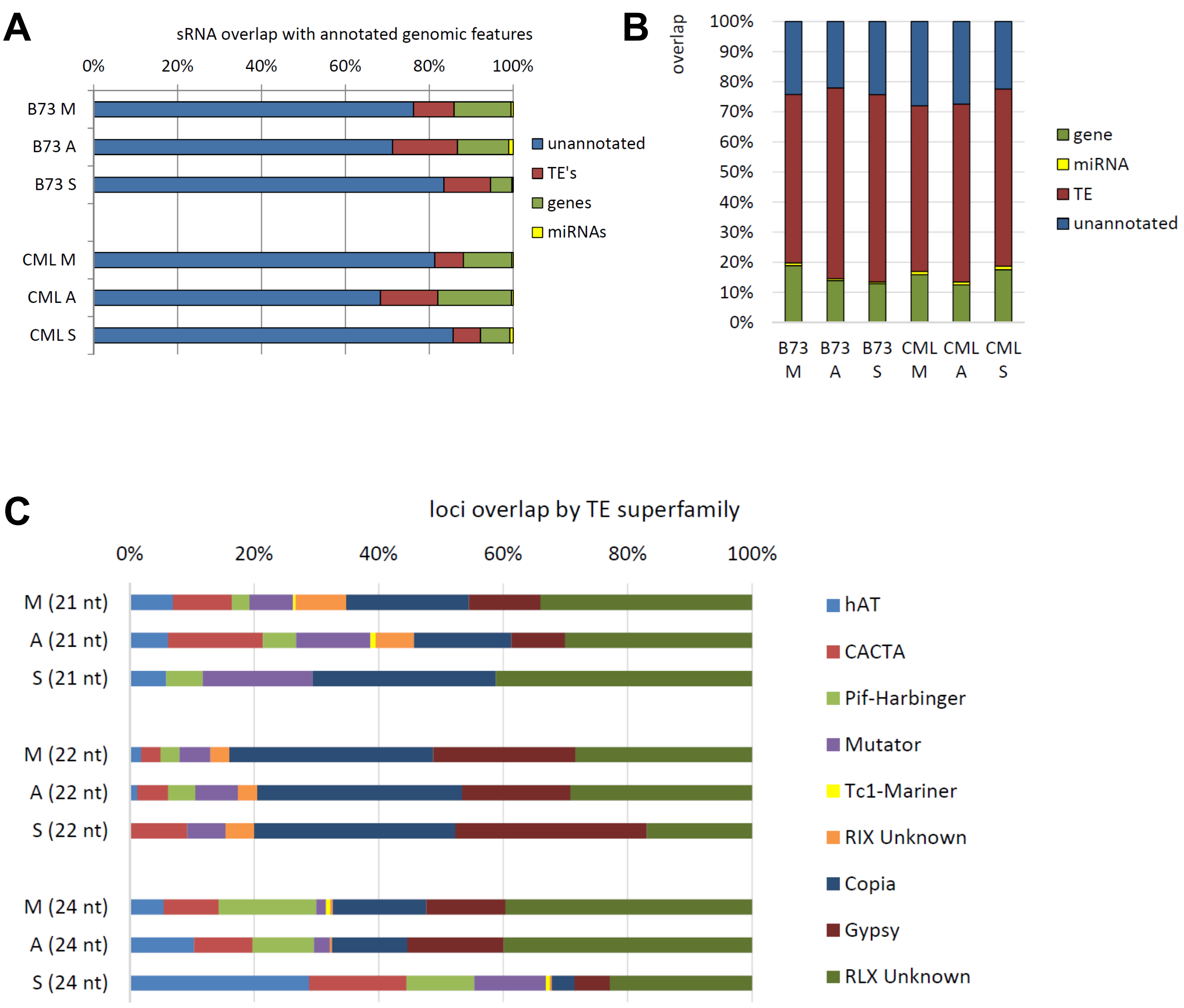

Supplementary Figure S1. Overlap of sRNA with annotated genome features
(A) Overlap of all sRNAs with genomic features (data from GSNAP alignment with maximum ambiguity = 85).
(B) Overlap of sRNA loci (with ≥ 2 RPM) with genomic features (data from butter alignment).
(C) Overlap of sRNA loci of specific nucleotide size (with ≥ 2 RPM) with genomic features (data from GSNAP alignment with maximum ambiguity = 5).
