# Supplementary material for: Novel Meiotic miRNAs and Indications for a Role of PhasiRNAs in Meiosis
Source: Front Plant Sci. 2016 Jun 2;7:762. doi: 10.3389/fpls.2016.00762 (PMC4889585; doi:10.3389/fpls.2016.00762)
Supplement: Supplementary file 4 [file Image_2.PDF]

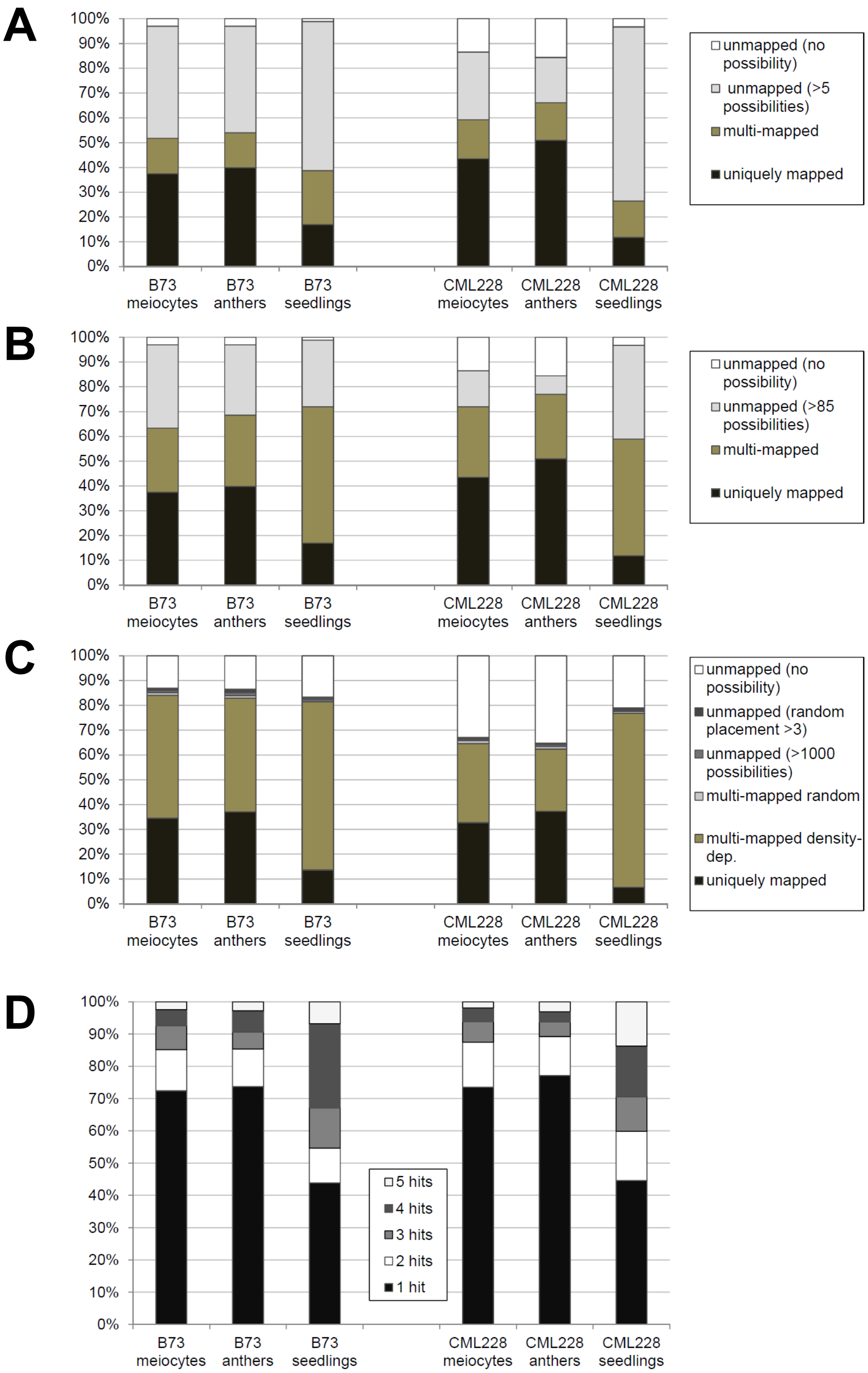

Supplementary Figure S2. Statistical analysis of obtained and retained sRNA reads

(A)-(C). Proportions of mapping outcome of obtained sRNA reads achieved with different algorithms for alignment (GSNAP with 5max_amb **(A)**, GSNAP with 85max_amb **(B)**, butter **(C)**).

(D) Aligned reads from (**A**), detailed into amount of multiple locations mapping to.
