# Supplementary material for: Novel Meiotic miRNAs and Indications for a Role of PhasiRNAs in Meiosis
Source: Front Plant Sci. 2016 Jun 2;7:762. doi: 10.3389/fpls.2016.00762 (PMC4889585; doi:10.3389/fpls.2016.00762)
Supplement: Supplementary file 5 [file Image_3.PDF]

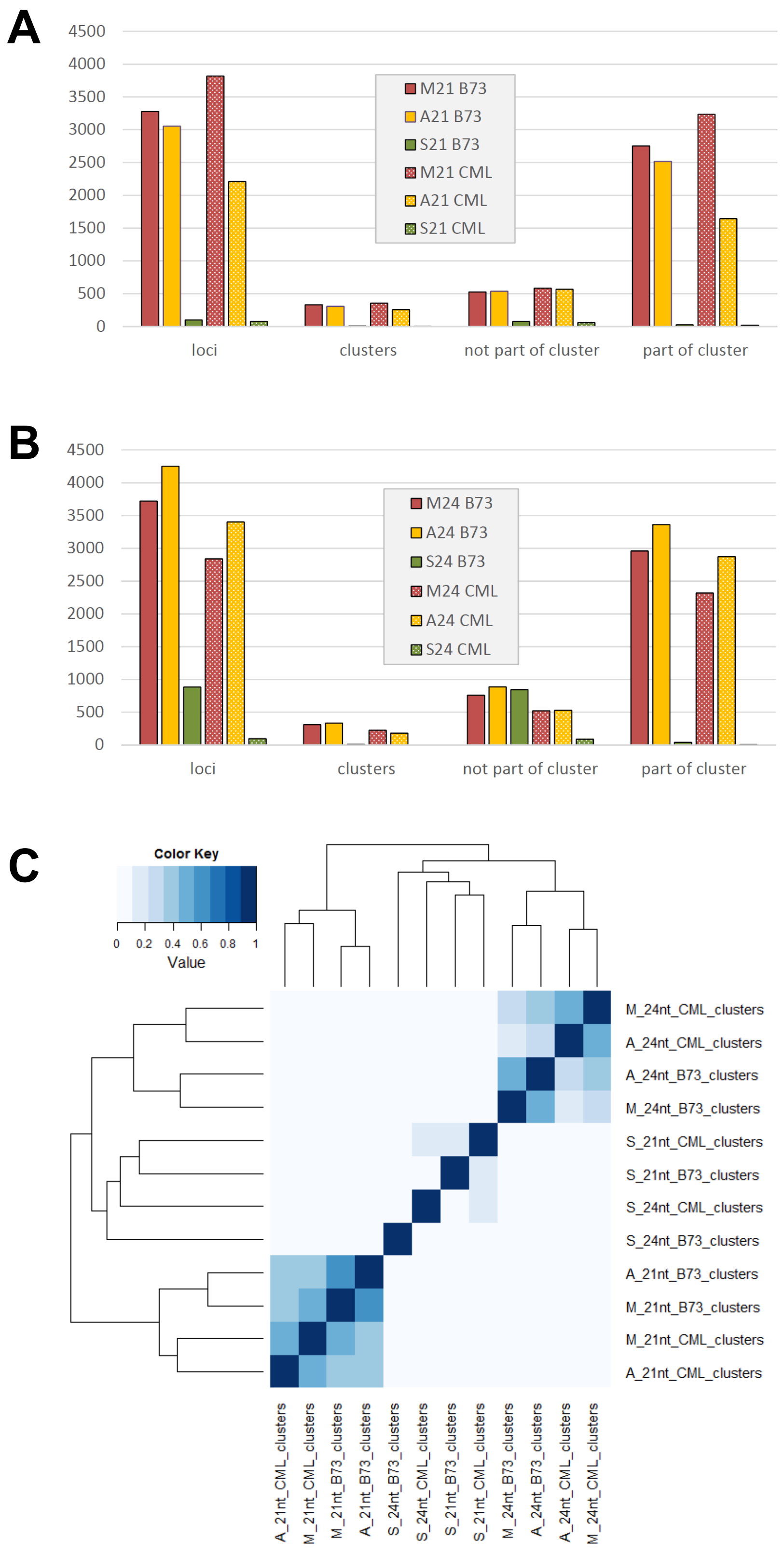

Supplementary Figure S3. sRNA clusters

(A)+(B). sRNA loci with at least 2 RPM, tested for being part of a cluster. Data for 21 nt sRNAs (**A**) and 24 nt sRNAs (**B**), in both B73 and CML228.

(C) Correlation heatmap of 21 nt and 24 nt clusters.

M = meiocytes, A = anthers, S = seedlings.
