# Supplementary material for: Novel Meiotic miRNAs and Indications for a Role of PhasiRNAs in Meiosis
Source: Front Plant Sci. 2016 Jun 2;7:762. doi: 10.3389/fpls.2016.00762 (PMC4889585; doi:10.3389/fpls.2016.00762)

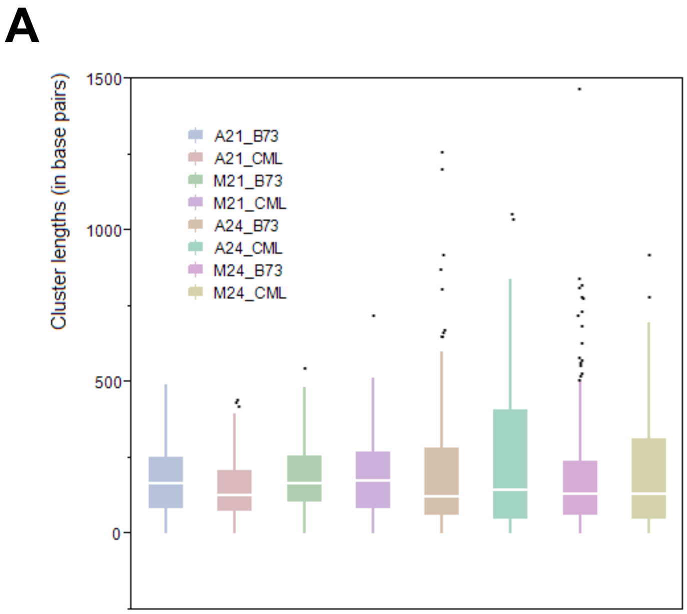

Supplementary Figure S4
Characterization of 21 nt and 24 nt cluster loci

(A) Boxplots of cluster lengths. Generated with JMP.

(B)-(E) Coverage plots of mean sRNA reads present at respective cluster loci in B73 meiocytes or anthers. Generated with bedtools.

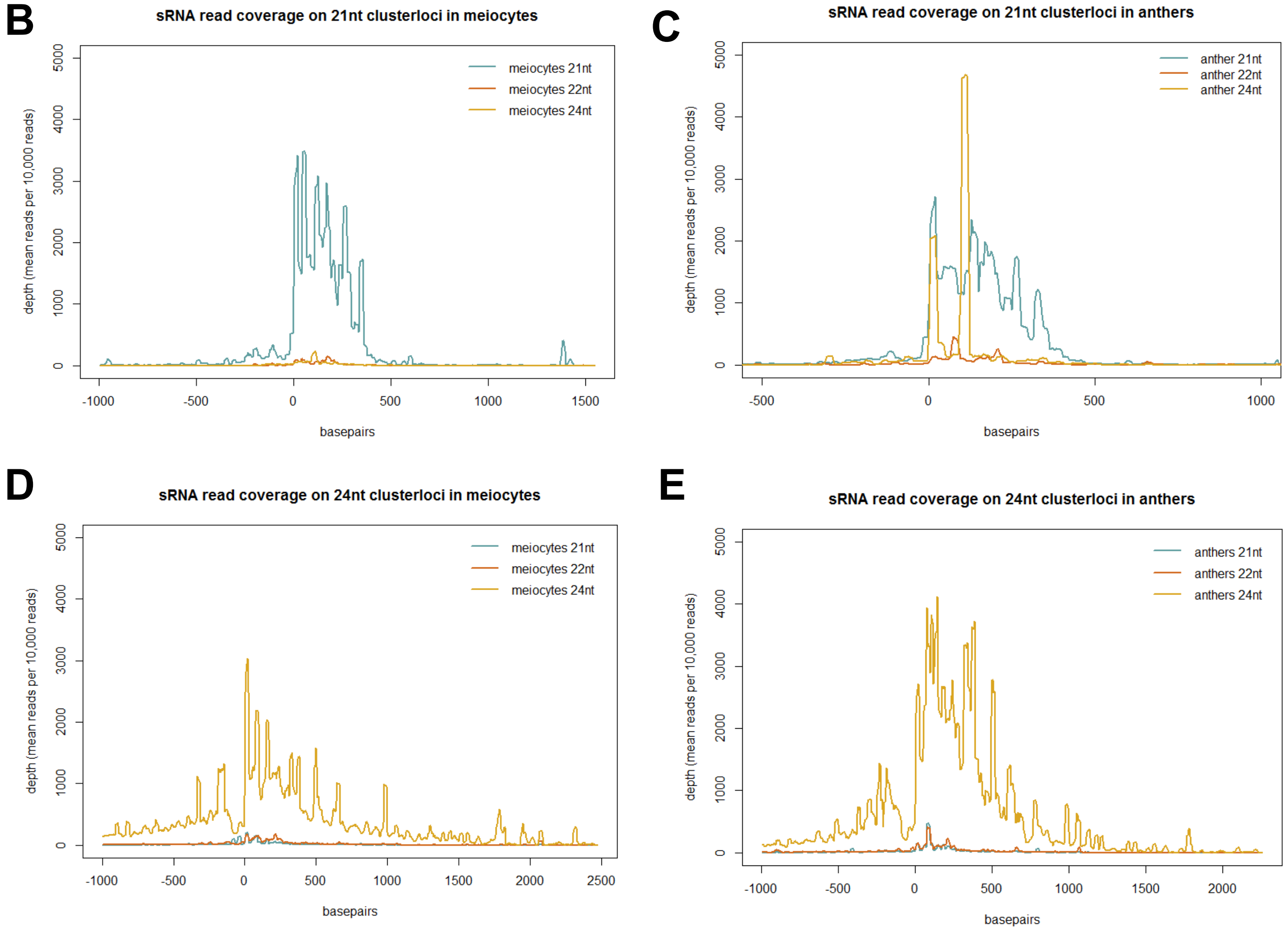

Supplement: Supplementary file 6 [file Image_4.PDF]
