# Supplementary material for: Novel Meiotic miRNAs and Indications for a Role of PhasiRNAs in Meiosis
Source: Front Plant Sci. 2016 Jun 2;7:762. doi: 10.3389/fpls.2016.00762 (PMC4889585; doi:10.3389/fpls.2016.00762)

Supplementary Figure S5. Genes overlapped by sRNA loci

(A) Venn diagram of genes overlapped by sRNA loci ≥ 2 RPM, shared or distinct for sample. Created with BioVenn.

(B) GO annotation of enriched genes in sRNA loci ≥ 2 RPM shared by B73 meiocytes and anthers. Created with AgriGO.

A

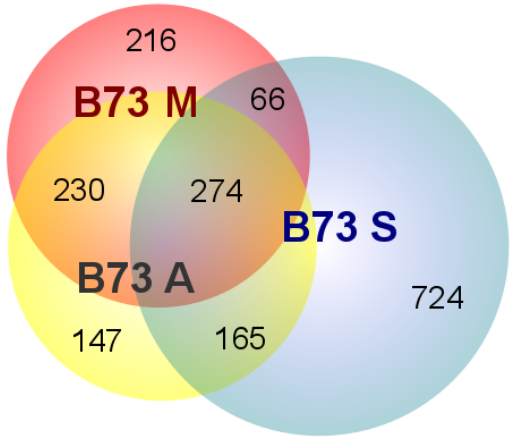

B

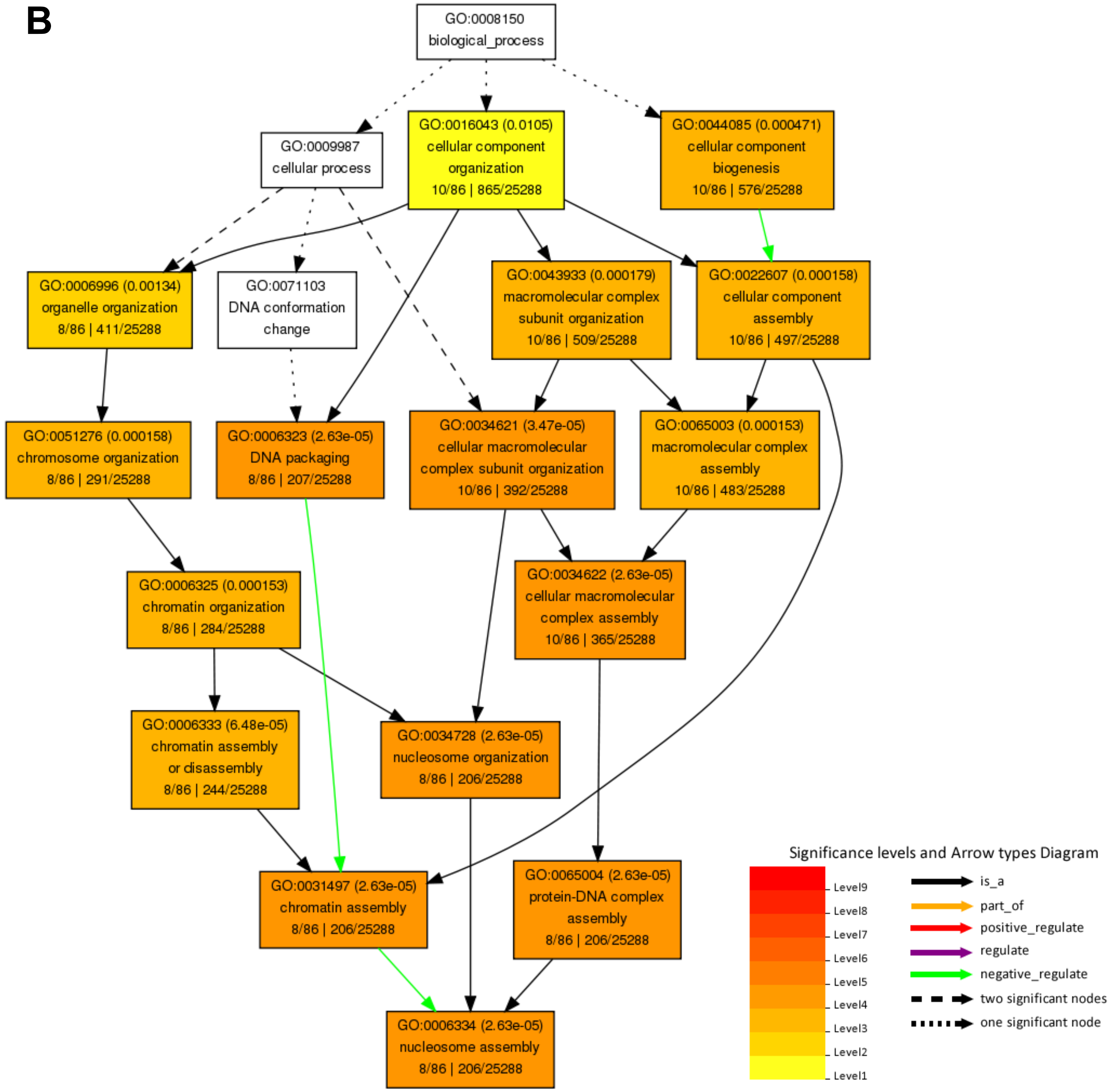

Supplement: Supplementary file 7 [file Image_5.PDF]
