# Supplementary material for: Novel Meiotic miRNAs and Indications for a Role of PhasiRNAs in Meiosis
Source: Front Plant Sci. 2016 Jun 2;7:762. doi: 10.3389/fpls.2016.00762 (PMC4889585; doi:10.3389/fpls.2016.00762)
Supplement: Supplementary file 8 [file Image_6.PDF]

- GO:0008150 biological_process
  - GO:0016043 cellular component organization [pv1:0.001 pv2:0.0013 pv3:1 pv4:1]
    - GO:0006996 organelle organization [pv1:0.049 pv2:0.00063 pv3:1 pv4:1]
    - GO:0022607 cellular component assembly [pv1:3.3e-06 pv2:8.8e-06 pv3:1 pv4:1]
    - GO:0006323 DNA packaging [pv1:0.00069 pv2:2.1e-06 pv3:1 pv4:1]
    - GO:0043933 macromolecular complex subunit organization [pv1:6.7e-07 pv2:2.1e-06 pv3:1 pv4:1]
  - GO:0009987 cellular process [pv1:1.4e-06 pv2:0.0012 pv3:0.00012 pv4:0.0027]
    - GO:0071103 DNA conformation change
    - GO:0006996 organelle organization [pv1:0.049 pv2:0.00063 pv3:1 pv4:1]
    - GO:0044237 cellular metabolic process [pv1:5.6e-05 pv2:0.0051 pv3:6.8e-06 pv4:0.0001]
    - GO:0034621 cellular macromolecular complex subunit organization [pv1:1.1e-06 pv2:2.5e-06 pv3:1 pv4:1]
  - GO:0008152 metabolic process [pv1:0.0035 pv2:1 pv3:7.6e-05 pv4:0.0047]
    - GO:0044238 primary metabolic process [pv1:0.018 pv2:1 pv3:0.0083 pv4:1]
    - GO:0044237 cellular metabolic process [pv1:5.6e-05 pv2:0.0051 pv3:6.8e-06 pv4:0.0001]
    - GO:0043170 macromolecule metabolic process [pv1:0.0071 pv2:0.02 pv3:0.016 pv4:0.025]
    - GO:0009058 biosynthetic process [pv1:0.00031 pv2:0.04 pv3:0.0009 pv4:0.0013]
    - GO:0044281 small molecule metabolic process
    - GO:0009056 catabolic process
    - GO:0055114 oxidation reduction [pv1:0.013 pv2:1 pv3:1 pv4:1]
  - GO:0051234 establishment of localization
    - GO:0006810 transport
  - GO:0051179 localization
    - GO:0051234 establishment of localization
  - GO:0044085 cellular component biogenesis [pv1:3.7e-05 pv2:8.3e-05 pv3:1 pv4:1]
    - GO:0070271 protein complex biogenesis [pv1:0.00069 pv2:1 pv3:1 pv4:1]
    - GO:0022607 cellular component assembly [pv1:3.3e-06 pv2:8.8e-06 pv3:1 pv4:1]

- GO:0003674 molecular_function
  - GO:0005215 transporter activity
  - GO:0005198 structural molecule activity [pv1:3.5e-19 pv2:1e-07 pv3:3.2e-16 pv4:1.2e-11]
    - GO:0003735 structural constituent of ribosome [pv1:9e-22 pv2:8.6e-12 pv3:3e-19 pv4:4.4e-16]
  - GO:0003824 catalytic activity
  - GO:0005488 binding

- GO:0005575 cellular_component
  - GO:0032991 macromolecular complex [pv1:2.6e-19 pv2:2.6e-16 pv3:6.9e-15 pv4:1.7e-09]
    - GO:0043234 protein complex
    - GO:0032993 protein-DNA complex [pv1:0.00069 pv2:1.9e-06 pv3:1 pv4:1]
    - GO:0030529 ribonucleoprotein complex [pv1:5.8e-22 pv2:8.6e-13 pv3:3.7e-19 pv4:2.2e-16]
  - GO:0005623 cell [pv1:5.6e-06 pv2:0.00042 pv3:0.0048 pv4:0.029]
    - GO:0044464 cell part [pv1:5.6e-06 pv2:0.00042 pv3:0.0048 pv4:0.029]
  - GO:0044464 cell part [pv1:5.6e-06 pv2:0.00042 pv3:0.0048 pv4:0.029]
    - GO:0044424 intracellular part [pv1:1.2e-08 pv2:4e-06 pv3:2.9e-07 pv4:0.00067]
    - GO:0016020 membrane
    - GO:0005622 intracellular [pv1:1.7e-08 pv2:5.7e-06 pv3:3.5e-06 pv4:0.0012]
    - GO:0044425 membrane part
  - GO:0043226 organelle [pv1:1.5e-08 pv2:7e-05 pv3:1.3e-06 pv4:0.0012]
    - GO:0043229 intracellular organelle [pv1:1.5e-08 pv2:7e-05 pv3:1.3e-06 pv4:0.0012]
    - GO:0043228 non-membrane-bounded organelle [pv1:3e-20 pv2:8e-14 pv3:1.8e-13 pv4:9.6e-10]
    - GO:0044422 organelle part [pv1:2.1e-06 pv2:7e-05 pv3:0.0046 pv4:1]
  - GO:0044422 organelle part [pv1:2.1e-06 pv2:7e-05 pv3:0.0046 pv4:1]
    - GO:0044446 intracellular organelle part [pv1:2.1e-06 pv2:7e-05 pv3:0.0046 pv4:1]

Supplementary Figure S6. Comparison of over-represented GO terms in sRNA overlapping genes

GO terms found significantly enriched in genes overlapped by sRNA read loci with ≥ 2 RPM. Analysis via AgriGO SEA comparison. pv1 = B73 M, pv2 = B73 A, pv3 = CML M, pv4 = CML A.
