# Supplementary material for: Novel Meiotic miRNAs and Indications for a Role of PhasiRNAs in Meiosis
Source: Front Plant Sci. 2016 Jun 2;7:762. doi: 10.3389/fpls.2016.00762 (PMC4889585; doi:10.3389/fpls.2016.00762)
Supplement: Supplementary file 9 [file Image_7.PDF]

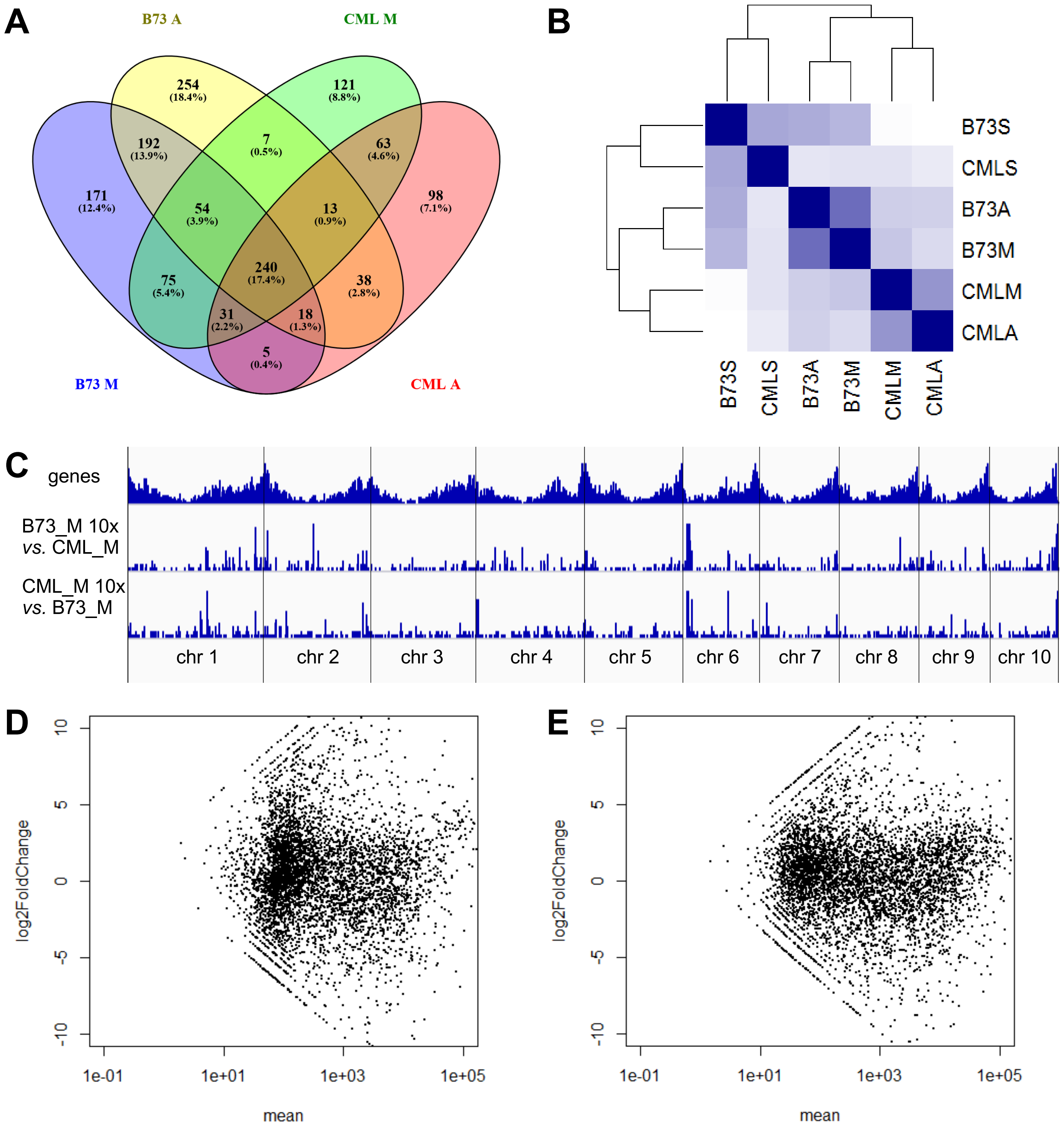

Supplementary Figure S7. Further comparison of sRNA data from B73 and CML.

A. Venn diagram of genes covered by ≥ 2 RPM sRNA reads (same sets of genes as used for GO analysis in Supplementary Figures 6 and 7). Created with DEseq.

B. Correlation heatmap of sRNA clusters (≥ 2 RPM).

C. sRNA loci with ≥10-fold difference between B73 and CML meiocytes. Plotted with IGV.

D. and E. MA intensity plots with average intensity (“A”) on x-axis and intensity ratio between B73 and CML samples (“M”) on y-axis, for meiocytes (**D**) and anthers (**E**). Created with DEseq.

M = meiocytes, A = anthers, S = seedlings.
